# Selection Maintains Photosynthesis in a Symbiotic Cyanobacterium Despite Redundancy With its Fern Host

**DOI:** 10.1093/molbev/msaf181

**Published:** 2025-08-06

**Authors:** Liam Friar, Kyle Keepers, Arkadiy I Garber, John P McCutcheon, Boswell Wing, Nolan C Kane

**Affiliations:** Department of Geological Sciences, University of Colorado, Boulder, CO, USA; Department of Ecology and Evolutionary Biology, University of Colorado, Boulder, CO, USA; Biodesign Institute and School of Life Sciences, Arizona State University, Tempe, AZ, USA; Biodesign Institute and School of Life Sciences, Arizona State University, Tempe, AZ, USA; Howard Hughes Medical Institute, Chevy Chase, MD, USA; Department of Geological Sciences, University of Colorado, Boulder, CO, USA; Department of Ecology and Evolutionary Biology, University of Colorado, Boulder, CO, USA

**Keywords:** molecular evolution, *Trichormus azollae*, symbiotic genome reduction, comparative genomics, genetic drift, *Azolla*

## Abstract

Vertically inherited symbionts experience different physical, chemical, and population genetic environments than free-living organisms. As a result, they can experience long-term reductions in effective population size (*N_e_*) and weaker purifying selection on genes that are less important in the host-associated environment. Over time, these forces result in gene loss. A comparative genomic approach using independently evolved symbiotic bacteria and free-living relatives can reveal which genes are important in the symbiotic state. We apply this approach to understand why some diazotrophic cyanobacteria evolving as vertically inherited symbionts of photosynthetic eukaryotic hosts have lost their ancestral capacity for photosynthesis while others have retained that capacity. We look specifically at *Trichormus azollae*, a diazotrophic cyanobacterium that remains photosynthetic after 50 to 90 Ma as a vertically inherited symbiont of *Azolla* ferns. We show that gene loss is ongoing, with different genes lost across the eight *T. azollae* strains examined. We apply molecular evolutionary models to genomes of *T. azollae* and free-living relatives, finding genome-wide signatures of drift in *T. azollae* consistent with long-term reductions in *N_e_*. Ribosomal proteins and proteins from the energy-capturing photosynthetic light reactions are under stronger purifying selection than genes from other pathways, including nitrogen fixation and photosynthetic carbon fixation. Strong purifying selection is expected for the ribosome given its extraordinary levels of conservation, even in ancient vertically inherited symbionts. That genes in the light reactions are under strong purifying selection and never lost in any strain suggests that energy capture, likely required for energy-intensive nitrogen fixation, remains important to this symbiont.

## Introduction

Symbioses span the tree of life (Rosenberg and Zilber-Rosenberg 2011; McCutcheon et al. 2024). They fill key ecological niches, including as the main sources of bioavailable nitrogen in many ecosystems (Davies-Barnard and Friedlingstein 2020; Zehr and Capone 2020; Tschitschko et al. 2024). Symbioses are also drivers of evolutionary complexity, including as the route by which eukaryotes have gained the capacities for photosynthesis and carbon fixation (McFadden 2001; Gabr et al. 2020). In some symbioses, the smaller partner (symbiont) is recruited by the larger partner (host) from the environment at each host generation, while in others, the symbiont is strictly passed from parent to offspring (“vertical inheritance”). When symbionts are vertically inherited there is often no free-living form of either partner. Vertically inherited symbionts lose ancestral cellular functions and, over time, can become obligately associated with their hosts (Moran et al. 2008). At the extreme, this loss of independent function and increase in host integration has resulted in symbiont-derived organelles including mitochondria, chloroplasts, and chromatophores (Gray and Archibald 2012).

Loss of cellular functions in symbionts is caused by the erosion and loss of functional genes (McCutcheon et al. 2024). In the canonical trajectory, initially, mobile elements proliferate in the symbiont genome and genes break, causing an accumulation of nonfunctional genes (pseudogenes) (McCutcheon and Moran 2012). Mobile elements and pseudogenes are later lost, such that the genome shrinks first in number of functional (intact) genes and then in total length (McCutcheon and Moran 2012). Gene loss can be adaptive, in which case the loss should be inevitable given the deletional bias in bacterial genomes and how easy it is for a mutation to “break” or “turn off” a gene (Giovannoni et al. 2014). Alternatively, neutral or even deleterious gene loss can occur due to probabilistic fluctuations in allele frequencies (drift) given the frequent population bottlenecks and lack of recombination that lead to persistent reductions in effective population sizes (*N_e_*) in vertically inherited bacterial symbionts (Mira and Moran 2002; Bobay and Ochman 2017).

While pseudogenization and gene deletion are pronounced in bacteria experiencing strong drift due to small *N_e_*, this, of course, is counteracted by selection for certain genes to remain functional (Bobay and Ochman 2017). Gene loss is determined, then, by the balance of drift and the selective advantage of one allele (here, a functional gene) over another (here, a lost or broken gene). Indeed, while there is strong evidence that many vertically inherited symbionts have very small *N_e_* (McCutcheon and Moran 2012), efficacious purifying selection is evident in the continued viability of symbionts over tens to hundreds of millions of years and patterns in which genes are maintained across independently evolving symbiont lineages (McCutcheon et al. 2024). Thus, genes retained by long-term symbionts reveal, sometimes in astonishing detail, which functions the symbionts perform in the host environment.

Cyanobacteria are an ancient and diverse group of bacteria that have formed symbioses with hosts from across the tree of life (Rai et al. 2000; Adams et al. 2013). Cyanobacteria and cyanobacteria-derived organelles perform the great majority of global carbon fixation and all oxygenic photosynthesis (Gray and Archibald 2012; Fischer et al. 2016; Crockford et al. 2023). Some cyanobacteria are also diazotrophic, fixing nitrogen on a globally significant scale (Warshan et al. 2016; Zehr and Capone 2020). Many of the most highly host-integrated vertically inherited symbionts and symbiont-derived organelles are descended from cyanobacteria that fix carbon or nitrogen for their hosts (Nakayama and Inagaki 2017; Gabr et al. 2020; Coale et al. 2024). Independent lineages of diazotrophic cyanobacteria have formed highly integrated, vertically inherited symbioses with multiple independent lineages of photosynthetic eukaryotic hosts (Becking 1987; Nakayama and Inagaki 2017; Nieves-Morión et al. 2023; Coale et al. 2024). In some of these, the symbiont (“diazo-cyanobiont”) has evolved to lose parts or all of photosynthesis, which comprises the energy-capturing “light reactions” and carbon-fixing Calvin–Benson–Bassham (Calvin or CBB) Cycle, living heterotrophically off of photosynthate from the host, while in others, the symbiont remains photosynthetic (Tripp et al. 2010; Nakayama and Inagaki 2017; Foster et al. 2022).

One diazo-cyanobiont that retains full photosynthesis is *Trichormus azollae*, a heterocystous cyanobacterium of the order Nostocales that lives as a vertically inherited symbiont of *Azolla*, a genus of aquatic ferns (Ray et al. 1979; Kaplan and Peters 1988, Ran et al. 2010). *T. azollae* is alternatively *Anabaena azollae* or *Nostoc azollae* (Pereira and Vasconcelos 2014); we use *Trichormus* in keeping with the trend in recent publications. *T. azollae* supplies *Azolla* with fixed nitrogen (Brouwer et al. 2017), and there is evidence that *Azolla* provides fixed carbon to *T. azollae* (Ray et al. 1979; Kaplan and Peters 1988), which means that *T. azollae* would have a host-derived source of reduced carbon were it to lose photosynthetic capacity. *Azolla* is unique among the hosts of the vertically inherited diazo-cyanobionts in being a land plant (Embryophyta) as opposed to an alga. In fact, *Azolla* is the only embryophyte known to have a strictly vertically inherited symbiont (not including plastids and mitochondria) (Rai et al. 2000; Adams et al. 2013). *T. azollae* is also unusual because it is one of the few known extracellular vertically inherited symbionts (Salem et al. 2015; Salem et al. 2017), living as populations within specialized compartments in the leaves of *Azolla* spp. (Peters and Mayne 1974; Ran et al. 2010; Li et al. 2018). Codiversification has been ongoing for ∼50 to 90 Ma (Metzgar and Pryer 2007; Testo and Sundue 2016; Li et al. 2018), and research on the first complete *T. azollae* genome found a large number of pseudogenes and low coding density, suggesting that *T. azollae* is at a well-described midpoint on the typical evolutionary trajectory of a vertically inherited symbiont (Ran et al. 2010; McCutcheon et al. 2019), where gene disruption has begun in earnest, but large-scale gene loss events have not culminated in a very small genome. Those genomic characteristics, along with observations of bottlenecks during symbiont propagation by the host (Becking 1987), suggest a small *N_e_*, although models of sequence evolution have not previously been applied to support or refute this idea.

Here, we use comparative genomic approaches to leverage 57 cyanobacterial genomes, including recently developed *dN/dS*-based models of sequence evolution to test the hypothesis of a small *N_e_* for *T. azollae*. We expand on previous analyses of gene loss and pseudogenization in *T. azollae*. We then determine which cellular functions are evolving under more drift-affected (less selection) or selection-dominated regimes. Because a gene can be affected by drift and weak selection even if its total functional elimination might be lethal (Moran et al. 2008), sensitive tests of the selective regime acting on intact genes can provide new information beyond inventories of pseudogenes and lost genes. Given the unique qualities of *T. azollae*, this analysis improves our understanding of what drives loss or retention of photosynthesis in diazo-cyanobionts, and of functions in vertically inherited symbionts generally.

## Materials and Methods

### Genome Annotations, Pseudogene Prediction, and Orthogroup Clustering

All sample accessions and abbreviations used are listed in supplementary table S1, Supplementary Material online. A total of 57 genomes are compared in this study. Our focal group is the *T. azollae* clade, for which we used eight genomes, including one complete reference-level genome from NCBI (*Nostoc Azollae* 0708, accession GCF_000196515.1; Ran et al. 2010) and seven contig-level metagenome-assembled genomes (MAGs) from ENA (project accession PRJEB45214; Dijkhuizen et al. 2021). A second set of *T. azollae* MAGs from Li et al. (2018) were not used because each of those strains was already included in the set from Dijkhuizen et al. 2021 (more detail in supplementary Material “Methods Sensitivity,” Supplementary Material online). Data from Song et al. (2025) were not used in order to avoid oversampling certain species of *Azolla* and because MAGs from individual samples were not available. We compared the symbiont ingroup to the genomes of 47 free-living Nostocales and two outgroup *Gloeobacter* genomes, which were obtained by searching for all complete “Nostocales” and “Gloeobacter” genomes on RefSeq (O’leary et al. 2016; Tatusova et al. 2016), released since 2010 January 1 using the command-line NCBI datasets tool (*ncbi-datasets-cli v. 15.6.0*; Sayers et al. 2022). Quality of MAGs was assessed using *checkM v1.0.18* (Parks et al. 2015) implemented in *KBase v1.4.0* (Arkin et al. 2018; Chivian et al. 2023 ).

Open reading frames (ORFs) were determined for all genomes by *prokka v. 1.14.6* (Seeman 2014), including for consistency the RefSeq genomes, which already had ORFs as annotated by the prokaryotic genome annotation pipeline (PGAP; Haft et al. 2018; Li et al. 2021). We determined that *prokka* ORF predictions were generally in strong agreement with RefSeq-predicted ORFs (supplementary Material “Methods Sensitivity,” Supplementary Material online) so we believe our results would be robust using the PGAP pipeline as an alternate annotation method.

Pseudogenes are genes that mutations have rendered nonfunctional. These can be identified based on homology to intact reference sequences. Pseudogenes were predicted by *Pseudofinder v. 1.1.0* (Syberg-Olsen et al. 2022). *T. azollae* genomes were run against a database of the intact genes from the 47 free-living Nostocales. The free-living Nostocales were run against the same database, but with each genome's own sequences removed. The *T. azollae* genomes were not included in the reference databases due to the high proportion of pseudogenes. All genes reported as pseudogenes in this manuscript were manually inspected to confirm the *Pseudofinder* prediction. We found that the number of genes predicted intact by *Pseudofinder* was on average within 2% of the number of intact genes as annotated on RefSeq (supplementary Material “Methods Sensitivity,” Supplementary Material online).

In order to determine gene loss and pseudogenization events among the taxa, as well as to perform sequence-based analyses of gene evolution, it was necessary to characterize all orthologous genes (orthologs) among the 57 taxa in this study. Groups of orthologs (orthogroups) were predicted using *OrthoFinder v. 2.5.5* (Emms and Kelly 2019). *OrthoFinder* uses a phylogenomic tree to improve orthogroup prediction. *OrthoFinder* generated an initial tree as part of its workflow, with concatenated gene alignment performed in *MAFFT v. 7.520* (Katoh and Standley 2013) and tree inference using maximum likelihood in *FastTree v. 2.1.11* (Price et al. 2010). The tree was then rooted on the two *Gloeobacter* genomes using the “*reroot*” function in *Gotree v. 0.4.3* (Lemoine and Gascuel 2021) and *OrthoFinder* was re-run with the rooted tree using the “*-ft*” flag. Except for the two *Gloeobacter* genomes, for which all ORFs were submitted, only ORFs that were predicted intact by *Pseudofinder* were used because of the possibility that the inclusion of pseudogenes would break the assumptions of *OrthoFinder*.

Seven of the free-living Nostocales genomes were deeply diverged; these and the two *Gloeobacter* genomes were removed from all subsequent analyses to maintain phylogenetic proximity between the *T. azollae* and the free-living Nostocales (supplementary fig. S1, Supplementary Material online). This left eight *T. azollae* and 40 free-living Nostocales genomes.

Predicted pseudogenes were assigned to orthogroups by *DIAMOND v. 2.1.7 blastx* (Buchfink et al. 2021) search with pseudogenes as queries and a database of all intact genes from the 40 free-living and eight *T. azollae* genomes. Pseudogenes were assigned to the orthogroup that contained the locus with the lowest *e-value* hit to the pseudogene and functional annotations were propagated from the orthogroup to the pseudogenes (see supplementary Material “Methods Sensitivity,” Supplementary Material online for more detail).

Functional annotation was performed by submitting all intact ORFs to eggnog-mapper online *v. 2.1.12* (Cantalapiedra et al. 2021) using default parameters. Gene names, gene descriptions, and KEGG numbers (Kanehisa and Goto 2000; Kanehisa 2019; Kanehisa et al. 2023) were taken directly from eggnog-mapper. Modules and pathways were determined by mapping KEGG numbers using the *KEGGREST v. 1.42.0* (Tenebaum and Bioconductor Package Maintainer 2023) package in *R* (R Core Team 2025). Annotations were propagated from loci to orthogroups inclusively: all annotations assigned to at least one locus in an orthogroup were propagated to the entire orthogroup. If functional annotations of different loci within an orthogroup disagreed with each other, that was noted (supplementary table S5, Supplementary Material online), but this was rarely the case and never the case for the loci discussed in the main manuscript.

Because the seven *T. azollae* MAGs are incomplete, genes could be falsely identified as absent or, in the case of an ORF that runs off the end of a contig, falsely identified as a pseudogene. This is not a systemic concern given the near completeness of the MAGs (*Fig. 2*; supplementary table S2, Supplementary Material online), and steps were taken to mitigate these shortcomings (supplementary Material Methods,” Supplementary Material online). In brief, MAGs were reassembled using different thresholds for read filtering and loci were considered intact if they were intact in any of the reassemblies from that sample.

### Strength of Selection

All of our strength of selection analyses are based on the ratio of the rate of selectively relevant nonsynonymous mutations (*dN*) to the rate of selectively neutral synonymous mutations (*dS*). The relative strength of selection acting on the *T. azollae* loci compared with orthologs in the free-living Nostocales was determined with *HyPhy RELAX v. 2.5.51(MP)* (Wertheim et al. 2015)*. RELAX* utilizes a branch-site model of sequence evolution to test the hypothesis that selection is more “intense” or more “relaxed” in an a priori ingroup of branches on a gene or species tree compared with an a priori outgroup (Wertheim et al. 2015). “Intense” selection means that there is a high probability that allele frequency will increase over generations for the fittest allele, while “relaxed” selection implies that allele frequencies are changing more randomly (Wertheim et al. 2015). Thus, “relaxed” selection could be the result of small *N_e_*, small |*s*|, or both (Charlesworth 2009). We want to emphasize that *RELAX* would call both stronger purifying selection (*dN/dS* << 1) and stronger directional selection (*dN/dS* > 1) “intensified selection”, despite the opposite nature of these two selective regimes. Moreover, and adding to the complex terminology, both purifying and directional selection could be present at different sites within the same gene (Wertheim et al. 2015). As such, *RELAX* does not test purifying versus directional selection—it only tests whether the balance of drift and selection is more tilted toward drift in the ingroup (“relaxed”) or more tilted toward selection in the ingroup (“intense”), relative to in the outgroup. Because we find it confusing to discuss “relaxed” selection (a function of *N_e_* and |*s*|) and simultaneously discuss the constituent |*s*| as “weak” or “strong” or any such adjective, in this manuscript, we replace “relaxed” with “drift-affected” and “intense” with “selection-dominated.” We also hope that these choices of terms emphasize that drift and selection are not mutually exclusive.

We ran *RELAX* on each orthogroup. The ingroup included all *T. azollae* terminal branches of the species tree as well as all internal branches that were ancestral to only the *T. azollae* clade. The outgroup consisted of all other branches. The root branch of the *T. azollae* clade was excluded from both groups because we do not know where along that branch the vertically inherited symbiosis began. To be included in this analysis, an orthogroup had to contain exactly one locus from each of at least three *T. azollae* genomes and at least three free-living Nostocales genomes. This threshold was a functional requirement for *RELAX* to create a distribution of *dN/dS* values. If a genome contained multiple loci assigned to a single orthogroup, none of those loci were included in the *RELAX* analyses because paralogs would break assumptions of the evolutionary model.

For each orthogroup included in this analysis, a multiple sequence alignment (MSA) was generated using *MACSE v. 2.07* (Ranwez et al. 2018). *MACSE* indicates possible frameshifts; this was very uncommon, and we determined that it was most straightforward to eliminate these sequences.

For each orthogroup analyzed with *RELAX*, the species tree was pruned to include only relevant genomes using the “*prune*” function in *Gotree*. The ingroup and outgroup were labeled using the “*rename*” function in *Gotree*. For each orthogroup, *RELAX* gave a *P*-value, which we corrected for multiple hypothesis testing with Benjamini and Hochberg (1995). Orthogroups were assigned results of “drift-affected,” “selection-dominated,” or “not significant” with corrected *P*_orthogroup_ < 0.1.

To determine if the number of drift-affected orthogroups was unevenly distributed among KEGG pathways, a new *P*_pathway_ was calculated for each pathway from a two-sided binomial test where the number of positives is the number of drift-affected orthogroups in that pathway, the number of tests is the total number of *RELAX* results for that pathway, and the hypothesized probability of success is the genome-wide portion of drift-affected orthogroups. These were corrected using Benjamini and Hochberg. Orthogroups were counted for each pathway to which they were assigned, and many were assigned to multiple pathways. The same analysis was performed for selection-dominated orthogroups. In addition to KEGG pathways, these analyses were performed for multicopy genes and orthogroups that contained pseudogenes. Four-letter gene names and KEGG KOs were the most granular functional annotations so if one of those annotations was assigned to multiple loci in the same genome, that indicated a multicopy gene. An orthogroup was considered multicopy for *T. azollae* if it was multicopy for at least one *T. azollae* genome.

In addition to looking at selection acting on individual orthogroups within the *T. azollae*, we were curious to see a single genome-wide comparison, to estimate the overall selective regime acting on the level of the organism. To that end, a concatenated alignment of all single-copy orthogroups was generated by concatenating the *MACSE* alignments for all orthogroups that had exactly one locus from each of the 48 genomes after elimination of sequences that contained frame-shift accommodations in the MSA. The species tree was used with ingroup/outgroup labeling as described above, but without pruning. Genome-wide *dN/dS* values were calculated using *hyphy-analyses FitMG94* (https://github.com/veg/hyphy-analyses/tree/master/FitMG94; Kosakovsky Pond et al. 2010) on this same concatenated alignment of single-copy orthogroups with the species tree.

**Fig. 1.**
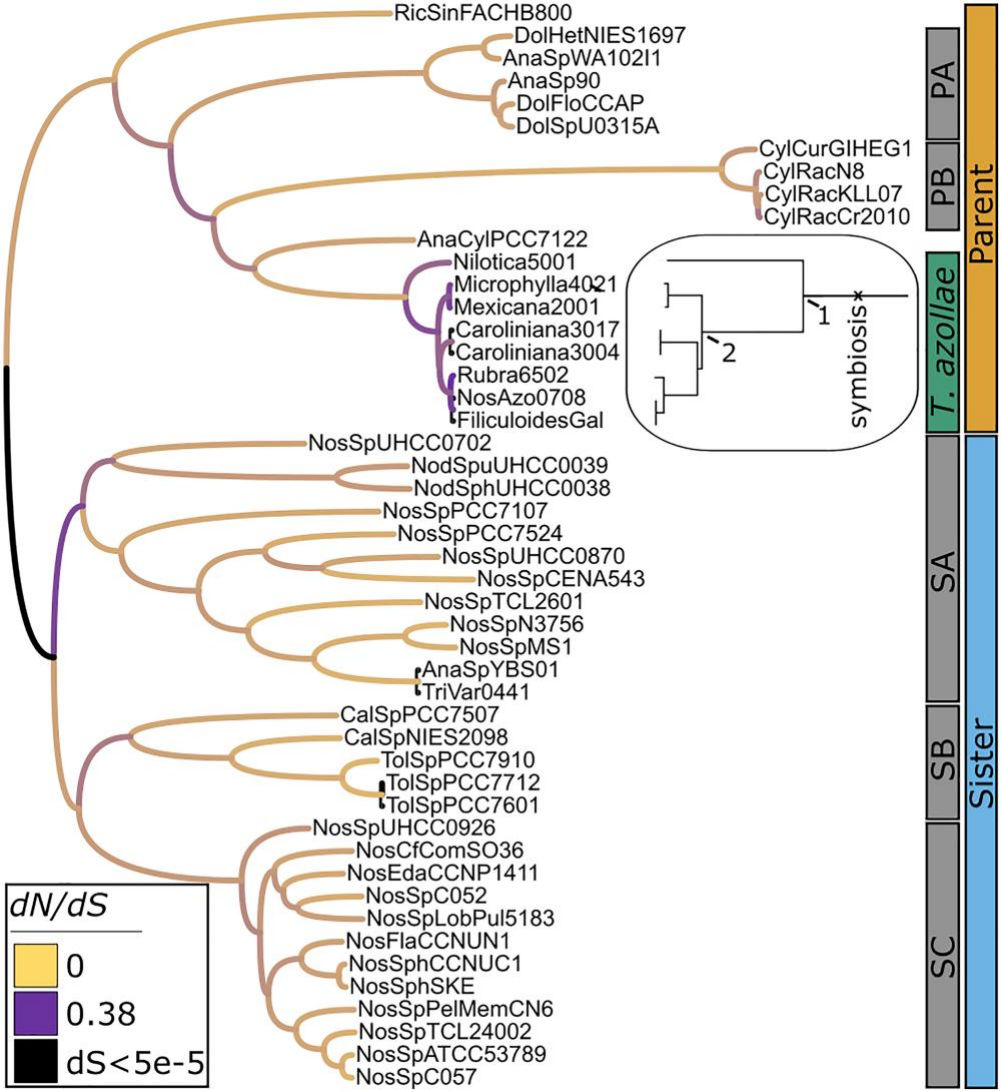
Genome-wide *dN/dS* on phylogenomic tree: Maximum likelihood species tree generated by *FastTree* implemented in *OrthoFinder*, rooted on the *gloeobacter* outgroups and pruned to include only the 48 taxa included in downstream analyses. Bootstrap values are one at each node. Focal *T. azollae* clade is labeled with green bar. Branches are colored by *dN/dS* calculated on a concatenated alignment of 1,015 single-copy *OrthoFinder*-generated orthogroups. Black branches have extremely low *dS* (<5e-5) so *dN/dS* calculations would be unreliable. All other branches have 5.1e-3 ≤ *dS* ≤ 1.1. “Parent” and “Sister” clades do not necessarily represent a meaningful biological partition but are useful for comparing the broader evolutionary context of the Nostocales to which *T. azollae* is most closely related to the rest of the Nostocales. The other subclades do not necessarily represent a meaningful biological partition but are useful for testing methods sensitivity as described in the main text and in Fig. 3. Inset: stretched view of *T. azollae* clade. Divergence times as estimated from Metzgar and Pryer 2007 and Testo and Sundue 2016: (symbiotic origin) 50 to 90 Ma; (1) ∼50 Ma; (2) ∼16 Ma.

Previous literature has established that *dN*/*dS* can be an unreliable signal of natural selection regimes at low *dS* (Mugal et al. 2014) and that comparing *dN*/*dS* across phylogenomic branches of different lengths may introduce biases where shorter branches are more likely to have elevated values (Wolf et al. 2009). We took three approaches to address possible biases introduced to our analyses of natural selection by the difference in distributions of branch lengths between *T. azollae* and the free-living Nostocales. First, we used different ingroups to repeat the process of running individual orthogroups through *RELAX*. The selection of different ingroups was based on clades that subjectively looked like the *T. azollae* clade in terms of total divergence and numbers of leaves (Fig. 1). For each ingroup, the outgroup was defined as the entire tree except the ingroup and the *T. azollae* clade, which was excluded to not skew the selective regimes in the outgroups. Second, we subsampled the genome set (removing four *T. azollae* and six free-living genomes) so that each pairwise distance between genomes had *dS* > 0.01 (supplementary fig. S5, Supplementary Material online) and then ran *RELAX* on the concatenated alignment of single-copy orthogroups with *T. azollae* as the ingroup as previously described for the full genome set, as well as calculating *dN*, *dS*, and *dN/dS* for the concatenated alignment of single-copy orthogroups along each branch in the subsampled tree. Third, we compared *dN/dS* values between *T. azollae* and the free-living Nostocales for only genomes with low *dS*.

### Other Analyses

A binomial test was also performed for enrichment of KEGG pathways for pseudogenes. Of necessity, only orthogroups that contained at least one pseudogene and had a KEGG pathway annotation were considered. In addition, orthogroups had to have loci present from at least six genomes (combined *T. azollae* and free-living) to parallel the thresholds for *RELAX*.

Supplementary table S5, Supplementary Material online includes the “Dates of minimum gene loss events” for each gene that is in the free-living Nostocales core, but not in the *T. azollae* core. These were determined using parsimony for the fewest total number of gene loss events, considering pseudogenes equivalent to absent genes.

## Results

### Initial Pipeline

A total of 57 genomes were obtained from public repositories (supplementary table S1, Supplementary Material online), of which, nine taxa were removed from the final analysis for being too phylogenetically distant for comparison to our ingroups. All following results and discussion refer only to the remaining 48 taxa (eight *T. azollae* and 40 free-living Nostocales). Though highly fragmented (264 to 368 contigs), the *T. azollae* MAGs are all highly complete (≥97.89% according to *checkM* cyanobacteria marker genes) and minimally contaminated (≤0.22%) (supplementary table S2, Supplementary Material online).

After ORF prediction with *prokka* and pseudogene prediction with *Pseudofinder*, a phylogenomic tree was generated using *FastTree* implemented in *OrthoFinder*, and rooted using *Gloeobacter* as the outgroup (Fig. 1). All bootstrap support values are one at each node. Some genera are not monophyletic, but that is to be expected given the difficulty of assigning genera within Nostocales (Huo et al. 2021; Mishra et al. 2021). *T. azollae* are monophyletic and their phylogeny matches the *T. azollae* phylogeny generated by Li et al. (2018), which used many of the same reference strains as our analysis but independently extracted DNA from different individuals. Notably, both our *T. azollae* phylogeny and that of Li et al. (2018) diverge from the host phylogeny of Li et al. (2018) in the placement of *A. caroliniana* and its symbiont. However, when a short internal branch is collapsed, the host and symbiont topologies agree. Although all bootstrap support values were one, given the importance of tree topology to selection analyses, we wanted to confirm the tree topology using an alternative method. To that end, we inferred trees using two alternative methods (*RAxML-ng*; Kozlov et al. 2019) using the same alignments as *FastTree* in *OrthoFinder* and *GTDB-tk* (Chaumeil et al. 2022), which uses a smaller set of marker genes, which increased our confidence in the tree that we used (see detailed discussion in supplementary Material “Methods Sensitivity,” supplementary fig. S1, and supplementary fig. S2, Supplementary Material online).

A KEGG identifier or four-letter gene name was assigned to 28.3% of orthogroups and 44.9% of loci, while KEGG pathways were assigned to 14.2% of orthogroups and 25.5% of loci. A small portion of loci were not placed into orthogroups by *OrthoFinder*. This represented 0.031% to 5.1% of intact sequences from the *T. azollae* genomes and 0.060% to 8.3% of intact sequences from the free-living genomes. The large majority of these sequences are poorly annotated and none of them affects any analyses presented in this manuscript.

We identify 1,042 “single-copy” orthogroups that each contain exactly one intact locus from each of the 48 genomes. After elimination of 27 such orthogroups in which *MACSE* detected frameshifts, alignments for the remaining 1,015 single-copy orthogroups were concatenated, generating one sequence per genome of 0.98 to 1.00 Mbp to be used in downstream analyses of single-copy orthogroups. This represents 30% to 33% of the orthogroups in each *T. azollae* genome and 27% to 31% of the loci in each *T. azollae* genome.

### Genome Statistics

**Fig. 2.**
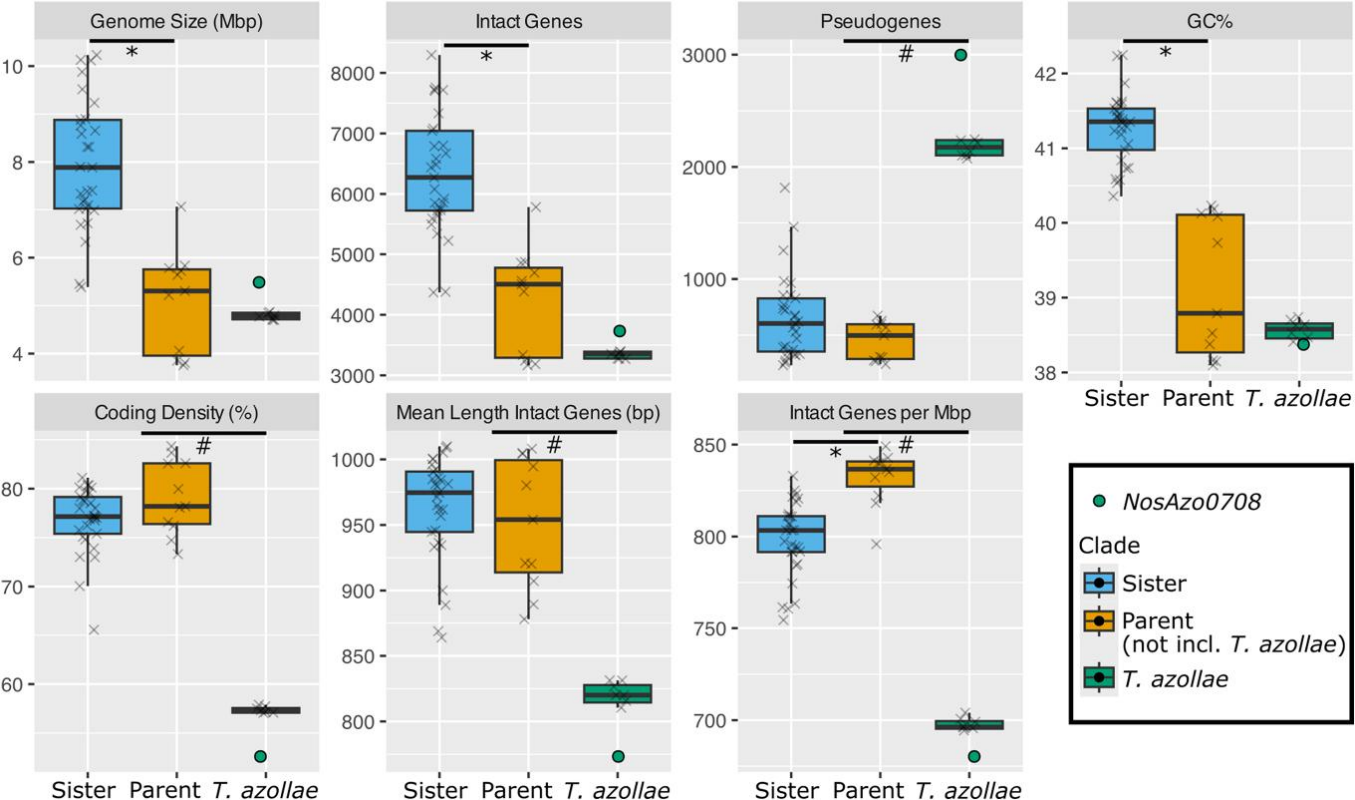
Genome statistics: genome statistics for 40 complete Nostocales genomes, one complete *T. azollae* genome, and seven *T. azollae* MAGs. Green circle represents *NosAzo0708*, the only complete *T. azollae* genome. Clades as labeled in Fig. 1. Parent clade does not include the *T. azollae* subclade. *: *P* < 10^−5^ (all others *P* > 0.05), Mann–Whitney–Wilcoxon Test for difference between Sister and Parent clades (not including *T. azollae*). #: value for *NosAzo0708* is greater or less than the range for all free-living Nostocales by >50% of the full free-living range.

The Parent clade genomes all have lower GC% than the Sister clade genomes and the Parent clade are also shorter with three exceptions (Mann–Whitney–Wilcoxon test *P* < 10^−5^ for each) (Fig. 2; supplementary table S3, Supplementary Material online). The only complete *T. azollae* genome, *NosAzo0708*, is within the free-living ranges in terms of GC%, genome length, and number of intact genes (Fig. 2; supplementary table S3, Supplementary Material online), although only the *Cylindrospermopsis*, which have the shortest genomes, have fewer intact genes.

All *T. azollae* are major outliers in terms of pseudogenes (Fig. 2), especially when accounting for genome length (supplementary fig. S3, Supplementary Material online). Counting pseudogenes, which are often fragmented, is difficult, but we are confident that this signal is real given its magnitude. Identifying mobile elements is outside of the scope of this study, but a significant number of mobile elements have previously been documented in *NosAzo0708* (Ran et al. 2010).

The 40 free-living Nostocales genomes show extremely strong linear correlation between genome length and number of intact genes (i.e. genes per Mbp, *R*^2^ = 0.99) and between genome length and the cumulative length of all intact genes (i.e. coding density, *R*^2^ = 0.97) (supplementary fig. S3, Supplementary Material online). *T. azollae* have the lowest coding density, due to having both the fewest intact genes per Mbp and the shortest mean length of intact genes (Fig. 2). The four *Cylindrospermopsis* have the highest coding density (supplementary table S3, Supplementary Material online).

### Core Nostocales Genes Missing From *T. azollae*

We define a core genome as genes that are intact in every genome within a given set of genomes, so, for instance, genes in the free-living core are intact in every free-living genome, but not necessarily in every *T.* azollae genome. The pan genome consists of all genes that are intact in at least one genome in a set of genomes, so the pangenome subsumes the core genome. For a summary of the core and pan genomes of *T. azollae* and of the free-living Nostocales as determined by orthogroups, see supplementary fig. S7, Supplementary Material online. We focus here on core and pan genomes as defined by functional annotations instead of orthogroups because this is more relevant to understanding which cellular functions might be compromised in *T. azollae*. So, for instance, if multiple orthogroups are annotated as *gene A*, no individual orthogroup has to be intact in every genome for *gene A* to be intact in every genome and thus part of the core genome. Thirty-nine genes (as identified by gene name and KEGG KO) from the free-living core were found to be pseudogenized or absent in at least one *T. azollae* genome. Some are highlighted here. All are summarized in supplementary table S5, Supplementary Material online, along with an estimated time since the functional gene was lost. We did not identify any functional annotations that were unique to *T. azollae*.

All of the *T. azollae* genomes contain a pseudogenized copy of *nifJ*, and no intact copies. *nifJ* is required for diazotrophic growth of *Anabaena sp. PCC 7120* only when iron is limited (Bauer et al. 1993). *moaB* and *moaC* are pseudogenes in all *T. azollae* genomes and *moaE* is additionally pseudogenic in *Nilotica5001*. The *moaABCDE* operon is involved in biosynthesis of the molybdenum cofactor MoCo, which is needed for nitrate reduction and all other functions that require a molybdenum catalyst except for nitrogen fixation, which requires a different molybdenum-bearing cofactor, FeMo (Rubio et al. 1998; Mendel 2013; Ringel et al. 2013). While the precise role of the heme oxygenase *hutZ/hugZ* (possibly intact in *Nilotica5001*, pseudogenic in all others) is not well established, it is involved in heme utilization, including heme degradation and possibly iron acquisition (Guo et al. 2008; Dojun et al. 2020). The vitamin B12 transporter *btuB* is absent from all *T. azollae* genomes. *T. azollae* genomes are compromised for sugar uptake including ABC sugar transporter proteins and a melibiose permease (*melB*, pseudogenic in all) that is responsible for glucose uptake and is ubiquitous among cyanobacteria (Moreno-Cabezuelo et al. 2019). Phosphorus acquisition is compromised, including alkaline phosphatases (*phoA*/*phoD*), a phosphonate transporter (*phnD*), and glycerophosphoryl diester phosphodiesterase (*glpQ*/*ugpQ*). Finally, *cheR*, a chemotaxis protein methyltransferase, is absent in all *T. azollae* genomes.

We also looked at some genes of interest including those involved in nitrogen fixation and photosystems I and II of the photosynthetic light reactions. All eight *T. azollae* have the same two orthologous copies of nitrogenase subunit *nifH* except for *Nilotica5001*, in which one of those copies is predicted to be a pseudogene due to an internal stop codon. The photosystem II gene *psbZ* is more than 50% longer in *T. azollae* than in the free-living Nostocales, but the sequences are highly conserved within the *T. azollae*, suggesting that this gene has diverged from the free-living form, but are still functional.

### Genome-wide Strength of Selection

A concatenated alignment of all single-copy orthogroups was used to calculate genome-wide *dN/dS* for each branch in the species tree (Fig. 1) and for a genome-wide *RELAX* model using *T. azollae* as the ingroup and free-living Nostocales as the outgroup. Eight branches have exceedingly low *dS* values (<5e-5) that we felt might be susceptible to noise in a *dN/dS* calculation. Removal of those eight branches leaves 85 branches with 5.1e-3 ≤ *dS* ≤ 1.1. Genome-wide *dN/dS* is elevated within the *T. azollae* clade: the 10 branches within the *T. azollae* clade (excluding the subclade root) have 10 of the 11 highest *dN/dS* values in the tree. The root of the *T. azollae* clade has a below-average *dN/dS* (69th highest of 85 branches). *RELAX* determines that the single-copy orthogroups are significantly more drift-affected within the *T. azollae* clade than in the free-living Nostocales (k = 0.50, *P* < 10e-4).

**Fig. 3.**
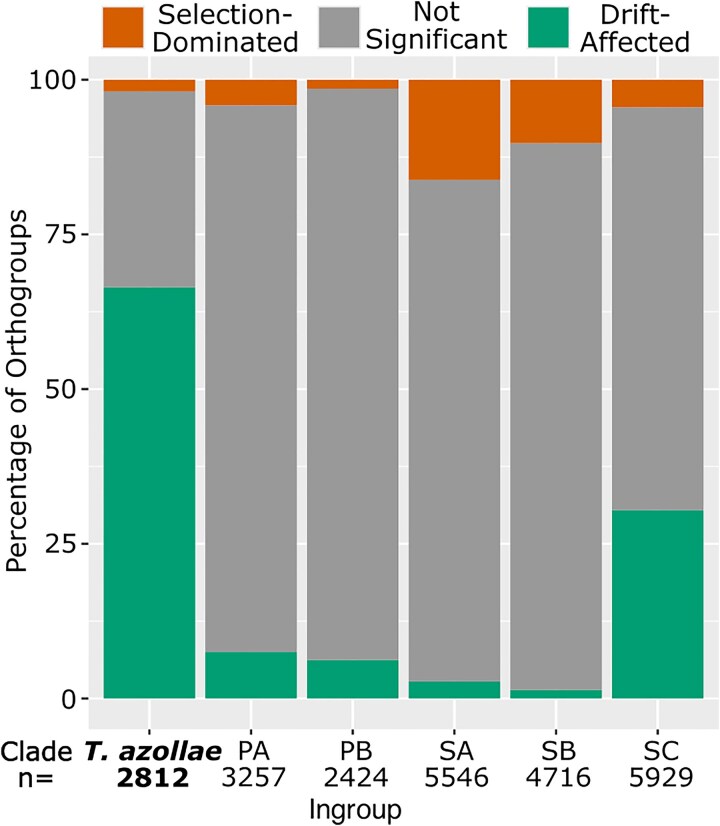
Percentage of orthogroups evolving under selection-dominated or drift-affected selective regimes: clades as shown in Fig. 1. Percentages of orthogroups with different *RELAX* results (corrected *P* ≤ 0.1) using different ingroups and using all noningroup branches except for *T. azollae* as the outgroup.

To improve the granularity of our understanding of selection in *T. azollae*, *RELAX* was run on individual orthogroups, again using *T. azollae* as the ingroup. Of the 2,825 orthogroups that met the thresholds described in *Methods*, 13 did not converge so were removed from downstream analyses. The remaining 2,812 orthogroups represent 81% to 85% of orthogroups and 73% to 82% of loci for the *T. azollae* genomes. Using a corrected *P*-value cutoff of *P* < 0.1 for each orthogroup, *RELAX* determined that 66.5% of orthogroups were significantly more drift-affected in *T. azollae* compared with in the free-living Nostocales, while 1.9% were more selection-dominated in *T. azollae*, and 31.7% did not differ significantly between ingroup and outgroup (Fig. 3).

We took three approaches to control for possible biasing effects of variable branch lengths in our selection analyses. Wolf et al. 2009 finds that *dN/dS* decreases in both mean and variance as *dS* increases, which is clearly replicated in our data, as visualized on graphs of genome-wide *dN/dS* versus *dS* for both the full genome set and a subsampled genome set for which the pairwise distance between any two genomes is *dS* > 0.01 (supplementary figs. S4a and S6a, Supplementary Material online). However, the *T. azollae* genomes clearly display elevated *dN/dS* even when compared only to free-living genomes of similar *dS* (supplementary figs. S4b and S6b, Supplementary Material online). *RELAX* run on the subsampled genome set determines that the single-copy orthogroups are significantly more drift-affected within the *T. azollae* clade than in the free-living Nostocales (*k* = 0.33, *P* < 10e-4). The gross results of the orthogroup-level *RELAX* results were essentially unchanged, with the percentage of drift-affected orthogroups in *T. azollae* changing from 66.5% to 66.3%, the percentage selection-dominated changing from 1.9% to 1.2%, and the percentage not significant changing from 31.7% to 32.5%. Finally, we repeated the individual orthogroup *RELAX* analysis using different ingroups (Fig. 3). We did not find any obvious bias toward one result over another. Three ingroups (including *T. azollae*) have more drift-affected orthogroups than selection-dominated orthogroups and three ingroups have the opposite. Clade SC has 30.4% drift-affected orthogroups, a much higher rate than the other four ingroups, which have 1.4% to 7.5% drift-affected orthogroups. Of the 12 strains in clade SC, four are nonsymbiotic, four are facultative symbionts of embryophytes, and four are facultative lichen symbionts (supplementary table S1, Supplementary Material online). In contrast, of the 28 strains outside of the *T. azollae* and SC clades, one is a facultative symbiont of an embryophyte, 23 are nonsymbiotic, and four are undetermined (supplementary table S1, Supplementary Material online). We still refer to facultative symbionts as free-living because they are free-living sometimes, unlike strictly vertically inherited symbionts, which are never free-living.

Decreasing the corrected *P*-value cutoff for significance decreases the percentage of significant results for each ingroup, but does not alter the main result that *T. azollae* has a much greater percentage of drift-affected orthogroups than do the other ingroups (42.0% for *T. azollae* at *P* < 0.01% and 26.6% at *P* < 0.001; supplementary table S4, Supplementary Material online).

### Evolutionary Trends in Individual Cellular Functions

**Fig. 4.**
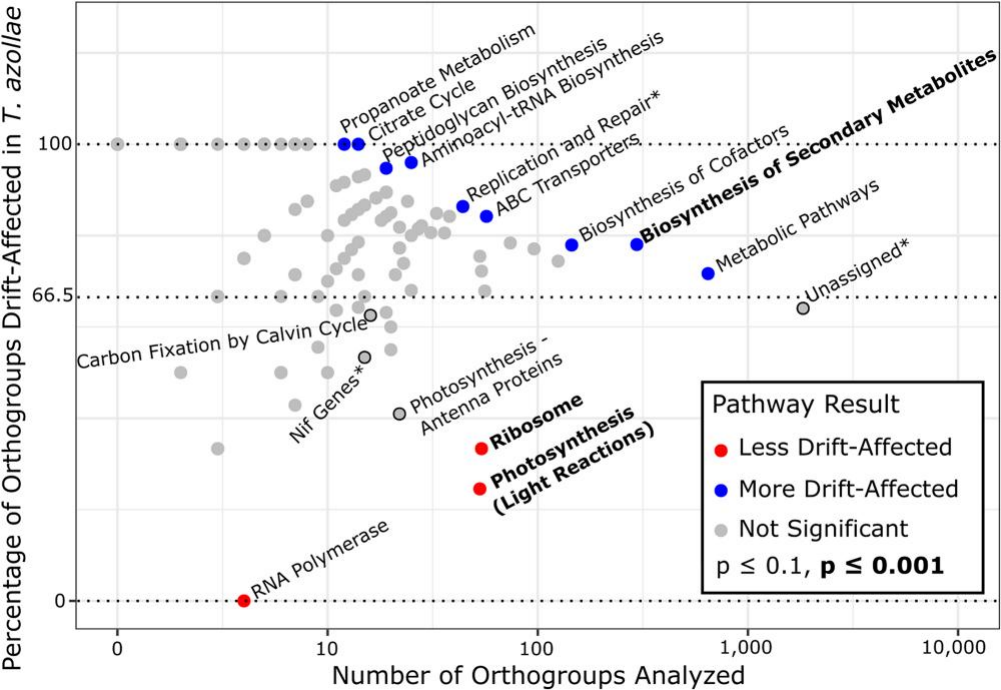
KEGG pathway enrichment for drift-affected orthogroups: orthogroups that are more drift-affected in *T. azollae* genomes than in the free-living Nostocales are nonrandomly distributed among cellular functions as defined by KEGG pathways. * are not KEGG pathways: “Replication and Repair” comprises five KEGG pathways that are grouped under this label on the KEGG database, “Nif genes” comprises all genes involved in nitrogen fixation with gene name abbreviations that begin with “*nif*” and “Unassigned” comprises all orthogroups not assigned to a pathway. A total of 982 orthogroups were analyzed. The central dashed line represents the genome-wide percentage of drift-affected orthogroups (66.5%). Pathways that differ significantly from that genome-wide percentage are labeled and are represented as blue and red circles. Pathways of interest with insignificant results are labeled and are represented as gray circles with black outlines.

Orthogroups affected by drift in *T. azollae* are nonrandomly distributed among KEGG pathways (Fig. 4 and Table 1). KEGG modules are subsets of KEGG pathways so could allow more granular analysis. However, most KEGG modules are so small that even extreme results (i.e. 100% or 0% drift-affected) would be insignificant; zero modules show significant divergence from the genome-wide percentage of drift-affected orthogroups. Though not significant, each of the four modules within the pathway “Photosynthesis” (light reactions) has fewer drift-affected orthogroups than the genome-wide average. Those modules are “Photosystem I,” “Photosystem II,” “Cytochrome b6f complex,” and “F-type ATPase, prokaryotes, and chloroplasts.” Similarly, though not individually significant, each of the five KEGG pathways grouped in “Replication and Repair” have more drift-affected orthogroups than the genome-wide average.

**Table 1:** KEGG pathway enrichment for drift-affected orthogroups: all pathways with a significant (corrected *P* ≤ 0.1) result from the analysis of enrichment of KEGG pathways for drift-affected orthogroups (OGs), as visualized in Fig. 4

	*P*	% Drift-Affected	# OGs Analyzed	# OGs NOT Analyzed
Lower percentage of drift-affected OGs than genome-wide percentage				
Photosynthesis (light reactions)	8.8e-08	25	53	40
Ribosome	3.7e-05	33	54	10
RNA polymerase	1.0e-01	0	4	5
Higher percentage of drift-affected OGs than genome-wide percentage				
Biosynthesis of secondary metabolites	4.8e-04	78	296	359
Aminoacyl-tRNA biosynthesis	2.3e-02	96	25	8
ABC transporters	5.4e-02	84	57	203
Citrate cycle	5.5e-02	100	14	9
Metabolic pathways	5.5e-02	72	646	965
Replication and repair^a^	6.1e-02	86	44	54
Peptidoglycan biosynthesis	6.2e-02	95	19	15
Biosynthesis of cofactors	6.8e-02	78	145	130
Propanoate metabolism	1.0e-01	100	12	10
Pathways of interest with nonsignificant results				
Photosynthesis—antenna proteins	1.6e-01	41	22	43
Unassigned^a^	1.7e-01	64	1830	14006
Nif genes^a^	4.3e-01	53	15	11
Carbon fixation by Calvin cycle	8.1e-01	62	16	7

^**a**^Are not KEGG pathways as described in Fig. 4 caption. A small set of pathways of interest with insignificant results are also shown. The lack of significant divergence in the “Unassigned” orthogroups from the genome-wide percentage of drift-affected orthogroups eases concerns that proteins with assigned functions in KEGG are biased toward particular evolutionary constraints. The category “% Drift-Affected” is based only on analyzed orthogroups. Orthogroups not analyzed were excluded as described in Methods.

No KEGG pathway or module showed significant divergence from the genome-wide proportion of selection-dominated orthogroups, and manual examination of functional annotations did not reveal any obvious patterns. Five of the 53 selection-dominated orthogroups were well-annotated and intact in all 47 genomes. Those five are: *folK* (involved in folate biosynthesis; Romine et al. 2017); *lgt/umpA* (lipid modification; Mao et al. 2016); *atpG* (involved in biosynthesis of ATP synthase; Chaux et al. 2023); *apcC* (involved in biosynthesis of phycobilisomes; Steiner et al. 2003), and *nifE* (part of the *NifNE* protein complex which is involved in biosynthesis of FeMo cofactor of nitrogenase; Fani et al. 2000).

There were 304 orthogroups that contained at least one *T. azollae* pseudogene that were also analyzed in *RELAX* (with pseudogenes removed from *RELAX* analysis, as described in *Materials and Methods*). The percentage of these that were drift-affected (66.8%) did not diverge significantly from the genome-wide percentage (66.5%). The percentage of these that were selection-dominated (4.9%, 15 orthogroups), however, was significantly greater than the genome-wide percentage (1.9%, uncorrected *P* = 7.8e-4). The 533 multicopy gene orthogroups that were analyzed in *RELAX* were significantly more likely to be drift-affected than the genome-wide rate (72%, uncorrected *P* = 4.4e-3).

We find that there is only one KEGG pathway that has significantly fewer pseudogene-containing orthogroups than the genome-wide proportion (using 19.7% of the 237 orthogroups that meet the thresholds described in *Materials and Methods*, not 35.0% of all orthogroups) and none that exceed it. That pathway is “Ribosome,” in which zero of 56 orthogroups contain a pseudogene from any *T. azollae* genomes (*P* = 9.7e-4) and all *T. azollae* genomes share the exact same set of orthogroups. All other pathways have corrected *P* > 0.35. Although not individually significant, if the two pathways involved in the light reactions, “Photosynthesis—antenna proteins” and “Photosynthesis” are combined, they have fewer pseudogenes than expected (*P* = 5.3e-2, five of 86 orthogroups). We acknowledge the small sample size and that pseudogene assignment to orthogroups and thus pathways was done with a less refined methodology than was used for intact loci.

## Discussion

We were motivated to understand the balance of drift and selection in shaping the ongoing genomic evolution of a vertically inherited symbiont. We were particularly interested in the selective regime under which the capacity for photosynthesis has been maintained in *T. azollae*, a diazotrophic cyanobacterial vertically inherited symbiont of the fern genus *Azolla*. We (i) confirm that loss of ancestral genes has occurred in *T. azollae* and that different strains have lost different genes, indicating that gene loss is ongoing; (ii) discover genome-wide effects of drift; (iii) discover that drift-affected genes are significantly nonrandomly distributed across cellular functions, which must indicate strong purifying selection on those functions that are less affected by drift, including the light reactions of photosynthesis. Thus, we answer both motivating questions: the *T. azollae* are evolving under the influence of both drift and selection, and photosynthesis is retained due to strong purifying selection on the light reactions. We explore each of these points in more detail in the sections below. At the end, we leverage our knowledge of other vertically inherited diazo-cyanobionts of photosynthetic eukaryotic hosts to propose that the heterocystous morphology of *T. azollae* is the reason that selection favors retention of photosynthesis.

### Absent and Broken Genes Indicate Compromised Cellular Functions

While photosynthesis has not been lost in *T. azollae*, other functions have. Here, we briefly discuss some of the genes that are core to all free-living Nostocales, but not to *T. azollae*. Knockout of *cheR* caused a loss of motility and host competency in the typically motile differentiated morphology (hormogonia) in a Nostocales that is a facultative symbiont of a different plant (Duggan et al. 2013). *T. azollae* lacks *cheR*, but its hormogonia are still motile (Adams et al. 2013). The loss of some phosphate and carbohydrate transporters has been noted in a previous genomic analysis of *T. azollae* (Ran et al. 2010); we expect, however, that there must remain some functional phosphate and carbohydrate transporters given the high ATP demands of nitrogen fixation (Inomura et al. 2020), evidence that *T. azollae* receives photosynthate from the host (Kaplan and Peters 1988), and genomic evidence of other carbohydrate transporters (Ran et al. 2010). Several missing or pseudogenized genes relate to uptake and utilization of metals. Biosynthesis of MoCo, which appears to be compromised, is required by many cellular functions, including nitrate reduction (Mendel 2013; Ringel et al. 2013). Indeed, *T. azollae* likely does not reduce nitrate (Holst and Yopp 1979; Ran et al. 2010). Interestingly, *nifE*, which is involved in biosynthesis of the FeMo cofactor of nitrogenase, the one molybdenum-bearing cofactor that does not require MoCo (Fani et al. 2000; Mendel 2013), is one of the few selection-dominated orthogroups in *T. azollae*. The pseudogenization of *nifJ* may indicate that the host ensures an adequate supply of iron to the symbiont. Biosynthesis of vitamin B12 (which contains cobalt) was lost in one, but not the other, of two vertically inherited diazo-cyanobionts of diatoms (Nakayama and Inagaki 2017), but potentially compromised B12 uptake suggests that this is not the case in *T. azollae*.

Further evidence for the loss of ancestral genes is revealed by low coding density in the *T. azollae* genomes, which is due in part to the large number of previously coding, but now noncoding pseudogenes. Low coding density and pseudogene accumulation are hallmarks of an intermediate stage in the canonical evolution of vertically inherited symbionts (McCutcheon and Moran 2012). Although the *T. azollae* have lost genes, their genomes are not (yet) superlatively reduced in terms of absolute number of intact genes relative to the free-living Nostocales: the *Cylindrospermopsis* (clade PB, also known as *Raphidopsis*), which are bloom-forming, freshwater, and planktonic (Aguilera et al. 2018), have the fewest intact genes. Strikingly, *T. azollae* have the lowest coding density while *Cylindrospermopsis* have the highest. While high coding density is observed in late-stage vertically inherited symbionts (McCutcheon and Moran 2012), in free-living bacteria like *Cylindrospermopsis*, it is more likely the result of selection for a streamlined genome, which could also explain the small number of intact genes in *Cylindrospermopsis* (Giovannoni et al. 2014).

*T. azollae* is not an outlier for GC% or genome length. While low GC% and small genomes are common characteristics of vertically inherited symbiont genomes (McCutcheon and Moran 2012; McCutcheon et al. 2019), including those that are extracellular (Salem et al. 2015; Salem et al. 2017), and of genomes from organisms with low *N_e_* more generally (Rocha and Feil 2010; Bobay and Ochman 2017), those are not universal correlations (Van Leuven and McCutcheon 2012).

Similar analyses were performed by Ran et al. (2010), and some of our findings confirmed their results, as cited above. However, Ran et al. (2010) used only one *T. azollae* genome (*NosAzo0708*) compared with our eight and their core genome was not Nostocales specific. Our broader taxonomic sampling of *T. azollae* reveals their diversity of gene content (supplementary table S5, Supplementary Material online). Placed in taxonomic context, these patterns of diversity give clues to the order of gene loss and reveal that gene loss is ongoing, with some core Nostocales genes being differentially lost on only one side of recently diverged *T. azollae* lineages (supplementary table S5, Supplementary Material online). The fact that photosynthesis has been retained across all eight independently evolving *T. azollae* lineages analyzed is thus all the more remarkable and strengthens the case that photosynthesis is maintained by strong purifying selection.

### Models of Molecular Evolution Reveal Genome-wide Drift Which Suggests Small *N_e_*

This is the first application of *dN*/*dS*-based molecular evolutionary models to *T. azollae* genomes. We find elevated *dN/dS* across a concatenated alignment of all single-copy orthogroups, which is characteristic of vertically inherited symbionts, suggests effects of drift, and generally indicates small *N_e_* (Bobay and Ochman 2017). Although elevated *dN*/*dS* can also indicate strong directional selection, this is unlikely to be the case genome-wide, as genome-wide histograms of *dN/dS* have a modal value close to zero (Buschiazzo et al. 2012). Drift is further confirmed by the *RELAX* model, which determines that the single-copy orthogroups are drift-affected. To determine if this signal is truly genome-wide, as opposed to being driven by a small subset of orthogroups, we ran *RELAX* on individual orthogroups with the result that 66.5% of orthogroups were drift-affected (corrected *P* < 0.1). The fact that 66.5% of orthogroups are drift-affected strongly suggests that *T. azollae* have a reduced *N_e_*: if the effects of drift were driven primarily by weak selection (low |*s*|) on individual genes, we would expect a more confined signal. To that point, even the KEGG pathway that is least drift-affected, “Photosynthesis,” still contains 25% drift-affected orthogroups.

The *T. azollae* have the lowest average gene length, which we believe supports that drift is driving gene loss: under pure drift, a mutation would be equally likely to fix at all loci, so longer genes, which provide a larger target for mutations, would be more likely to accrue mutations and break faster. Although *T. azollae* is not experiencing pure drift, many genes are experiencing significant drift such that this mechanism may be relevant.

Graphs of *dN/dS* by *dS* (supplementary fig. S4a and S6a, Supplementary Material online) clearly demonstrate the potential for biases when comparing *dN/dS* among branches of greatly different *dS*. However, the central results that *T. azollae* have an elevated genome-wide *dN/dS*, that the concatenated alignment of single-copy orthogroups is drift-affected, and that ∼66.5% of orthogroups are drift-affected (*P* ≤ 0.1) are replicated when comparing only to similarly short *dS* branches of the free-living Nostocales (supplementary fig. S4b and S6b, Supplementary Material online) and when subsampling the genome set so that all pairwise distances between genomes have *dS* > 0.01. Use of alternative ingroups demonstrates that the orthogroup-level *RELAX* results are not biased toward drift-affected in every case where the ingroup is significantly less diverged than the outgroup. The sensitivity of this analysis is further supported by the fact that clade SC, which contains facultative symbionts, has a percentage of drift-affected orthogroups that is intermediate between *T. azollae* and the strictly free-living clades, considering that facultative symbionts can exhibit similar genomic signatures to vertically inherited symbionts, though less extremely (Moran et al. 2008). While we cannot rule out some effects of the biases introduced by uneven branch lengths between in and outgroup, we believe that these controls demonstrate the central results of these analyses should be robust to any biases.

### Drift-affected Orthogroups are Significantly Nonrandomly Distributed Among Cellular Functions, Indicating Differences in Strength of Selection

Due to the lack of recombination in genomes of other vertically inherited symbionts (McCutcheon et al. 2019), we assume that *N_e_* is the same for all loci in the *T. azollae* genome (Charlesworth 2009). Thus, any differences among cellular functions in the balance of drift and selection must be due to different strengths of selection (Charlesworth 2009). While the *RELAX* model does not specify purifying selection, an intact gene must be under purifying selection at least to the degree that nonfunctional alleles have been selected against.

Several pathways have a significantly elevated percentage of drift-affected orthogroups, indicating weak purifying selection on these pathways (Fig. 4). The identities of these pathways suggest that genes with more confined effects experience weaker selection on average: secondary metabolites are peripheral to central metabolites and “Metabolic pathways” at large might have fewer network effects than the ribosome, for instance. Contrary to that pattern, all of the genes within the “Propanoate metabolism” pathway are also involved in other pathways of central carbon metabolism including pyruvate metabolism and the citrate cycle. Weak selection on those genes as well as the “Citrate cycle” is interesting given the unusual and poorly understood carbon economy of this symbiosis. We note that cyanobacteria generally lack the complete canonical citrate cycle (Zhang and Bryant 2011).

Weak selection on “Biosynthesis of cofactors” and “ABC transporters” parallels some of the missing genes discussed earlier. Poor DNA repair is a hallmark of vertically inherited symbionts (Moran et al. 2008) and degradation of both replication and repair machinery may facilitate the accumulation of mutations that cause pseudogenes and rapid molecular evolution (Moran et al. 2008; McCutcheon et al. 2024). Peptidoglycan biosynthesis is lost in several intracellular vertically inherited symbionts of insects (McCutcheon and Moran 2012); genes involved in peptidoglycan synthesis may also be involved in heterocyst development (Videau et al. 2016). The significantly elevated rate of drift-affected orthogroups among multicopy genes is in line with what we know about evolution in multicopy genes (Copley 2020); we do not think this phenomenon affects our pathway-level results because such multicopy genes are not obviously over- or under-represented in the KEGG pathways.

There are very few selection-dominated orthogroups and no significant signal in their distribution. This makes sense given that *RELAX* uses free-living Nostocales to set the null expectation, so the strength of selection would have to be so much stronger in *T. azollae* to overcome small *N_e_*.

### Ribosomal Proteins and the Light Reactions of Photosynthesis Experience the Strongest Selection

The KEGG pathways “RNA polymerase” (subunits of that enzyme) and “Ribosome” (ribosomal proteins) have significantly lower percentages of drift-affected orthogroups, indicating strong purifying selection. This makes sense given the central importance of transcription and translation to all cellular functions and the fact that these functions are not easily outsourced to a host. It also matches expectations from other vertically inherited symbionts: genes encoding ribosomal proteins, subunits of RNA polymerase, and aminoacyl-tRNA synthetases are among the few protein-coding genes that are generally intact even in the smallest vertically inherited symbiont genomes (Tripp et al. 2010, Graf et al. 2021; Rangel-Chávez et al. 2021; McCutcheon et al. 2024). This result thus increases our confidence in our approach. Paradoxically, given that tRNAs work in tandem with ribosomes, orthogroups in “Aminoacyl-tRNA biosynthesis,” which consists mainly of tRNA synthetases, are marginally significantly more likely to be drift-affected than the genome-wide average. Loss of sequence conservation has previously been observed in aminoacyl-tRNA synthetases of parasites and vertically inherited symbionts, specifically in the editing domains (Melnikov et al. 2018). We did not perform domain-level analyses, so that should be explored in future work.

“Photosynthesis” (light reactions), appears to be under the strongest purifying selection. The results for “Carbon Fixation by Calvin Cycle” and for the *nif* genes (nitrogen fixation), however, are not significant, indicating that they are not under particularly strong purifying selection. If organismal fitness is proportional to metabolic flux and selection is strongest on flux-limiting steps (Dykhuizen and Dean 1990), these results suggest that this system is more often limited not by carbon and nitrogen fixation themselves, but by the light reactions, which generate cellular energy and reducing power for carbon and nitrogen fixation (Magnuson 2019). Remembering that these results are in comparison to free-living Nostocales, this makes sense if *T. azollae* is receiving some amount of fixed carbon from the host (Kaplan and Peters 1988) and the symbiosis is not growth limited by nitrogen fixation (Brouwer et al. 2017). Our hypothesis is obviously not that nitrogen fixation is not important to the symbiosis, but that the light reactions are the rate-limiting steps. Likewise, carbon fixation may be important but not as important as the light reactions. Alternatively, carbon fixation may be maintained to facilitate the light reactions and not because carbon fixation is itself important (Thornton 2014; Braakman et al. 2017). “Glycolysis (Embden–Meyerhof pathway)” and “Pentose phosphate, oxidative phase” are KEGG modules that are involved in converting photosynthate to cellular energy and reductants, respectively (Magnuson 2019); as with all other modules, neither differed significantly from the genome-wide percentage of drift-affected orthogroups.

The importance of ribosomes and the light reactions are supported by other analyses. Although we find no general correlation between an orthogroup containing pseudogenes and being drift-affected, ribosomal proteins and proteins from the combination of “Photosynthesis” and “Photosynthesis—antenna proteins” are significantly less likely to be pseudogenes. Furthermore, none of the missing Nostocales core genes belong to either of these functions.

We have thus answered our motivating questions: the *T. azollae* genomes are evolving under the influence of both drift and selection, and strong purifying selection on the light reactions drives the retention of photosynthesis.

### Evolutionary Convergence of Cyanobacterial Symbionts Depends on Compartmentalization of Photosynthesis and Nitrogen Fixation

The *Azolla-T. azollae* symbiosis is one of at least four independently evolved vertically inherited symbioses between a diazotrophic cyanobacteria and a photosynthetic eukaryotic host. This allows a comparative approach to understanding why photosynthesis experiences strong purifying selection in *T. azollae* when photosynthesis has been lost in some of these comparable symbionts. Unlike *T. azollae*, the other three symbionts, *UCYN-A*, spheroid bodies, and *Richelia euintracellularis*, live intracellularly within non-Viridiplantae algal hosts (haptophyte algae including *Braarudosphaera bigelowii*, diatoms of the family Rhopalodiaceae, and *Hemiaulus hauckii*, respectively) (Tripp et al. 2010; Hilton et al. 2013; Nakayama and Inagaki 2017; Caputo et al. 2019; Nieves-Morión et al. 2023). All of these symbionts, including *T. azollae*, appear to receive photosynthate from their hosts (Ray et al. 1979; Kaplan and Peters 1988; Nakayama et al. 2014; Foster et al. 2022). While precise dating of the onsets of these symbioses is difficult, all are thought to be in the range of ∼50 to 100Ma (Metzgar and Pryer 2007; Cornejo-Castillo et al. 2016; Testo and Sundue 2016; Caputo et al. 2019), except for spheroid bodies, which might be as young as ∼25 Ma (Caputo et al. 2019) or even ∼12 Ma (Nakayama et al. 2011). *R. euintracellularis* and *T. azollae* retain full photosynthesis while spheroid bodies have lost photosystem I, photosystem II, and carbon fixation, and *UCYN-A* retains only photosystem I (Ray et al. 1979; Kaplan and Peters 1988; Jahson et al. 1995; Zehr et al. 2008; Nakayama and Inagaki 2017; Nieves-Morión et al. 2020; Flores et al. 2022).

Strikingly, *R. euintracellularis* and *T. azollae*, both of which retain photosynthesis, are heterocystous Nostocales, while *UCYN-A* and spheroid bodies, each of which have lost at least photosystem II and carbon fixation, are unicellular representatives of other cyanobacterial clades. The fact that retention of photosynthesis by the symbiont correlates with whether the symbiont is a heterocystous Nostocales or unicellular, and does not correlate with whether the host is a multicellular land plant or a unicellular alga, nor with the location of the symbiont relative to the host (intracellular vs. extracellular), strongly indicates that retention of photosynthesis is at least partly determined by whether the symbiont is a heterocystous Nostocales. Importantly, other heterocystous Nostocales that form facultative symbioses with embryophytes fully down-regulate photosynthesis to live heterotrophically off photosynthate from the host (Rai et al. 2000). This suggests that loss of photosynthesis would not be fatal for *T. azollae* and *R. euintracellularis* and that there must be a selective advantage to retaining photosynthesis that does not exist for unicellular symbionts.

We believe that the presence of heterocysts in *T. azollae* and *R. euintracellularis* has facilitated the retention of photosynthesis. Nitrogenase, the enzyme that fixes nitrogen, is extremely sensitive to O_2_, so diazotrophic cyanobacteria must separate nitrogen fixation from photosynthesis, particularly O_2_-evolving photosystem II and carbon fixation to which it is closely tied (Kumar et al. 2010). Heterocystous cyanobacteria separate the two processes spatially, with nitrogen fixation occurring in specialized cells called heterocysts that lack carbon fixation and active photosystem II (Zeng and Zhang 2022). Unicellular diazotrophic cyanobacteria instead achieve separation temporally by fixing nitrogen only at night (Kumar et al. 2010). Thus, there may be a selective advantage to losing photosystem II and carbon fixation that is unique to a unicellular diazo-cyanobiont: relying solely on photosynthate from the host allows them to fix nitrogen during the day when the host is photosynthesizing. Indeed, *UCYN-A* and spheroid bodies fix nitrogen during the day (Gradoville et al. 2021, Abresch et al. 2024) and *UCYN-A* maintains photosystem I which does not generate O_2_ (Zehr et al. 2008). Diurnal nitrogen fixation is advantageous because it allows direct transfer of ATP and reductant from photosynthesis, unlike nocturnal nitrogen fixation, which requires photosynthate to be stored and later broken down (Scherer et al. 1988). Additionally, the lack of differentiated cell types in the unicellular diazo-cyanobionts may facilitate the transition to a simpler, nitrogen-fixing pseudo-organelle compared with a filamentous Nostocales, which, for instance, requires vegetative cells to reproduce but those vegetative cells cannot fix nitrogen (Zeng and Zhang 2022).

This comparative analysis suggests that the inability of unicellular cyanobacteria to spatially separate nitrogen fixation and photosynthesis within themselves creates a selective advantage, possibly in terms of the cellular energy economy, to separating those processes by losing photosynthesis and outsourcing it to the host. Our molecular evolutionary analysis revealed strong purifying selection acting on the light reactions of photosynthesis in *T. azollae*, suggesting that *T. azollae* is most often limited for cellular energy and reducing power, which feed both carbon and nitrogen fixation. More work is needed to develop detailed models of these tradeoffs to understand the optimum, most efficient metabolic strategies of these symbionts, as well as to quantify the symbionts' photosynthetic metabolism under various conditions.

## Supplementary Material

msaf181_Supplementary_Data

## Data Availability

Accessions for all genomes used, including MAGs, are in supplementary table S1, Supplementary Material online. Specific commands used for different programs are available in supplementary methods, Supplementary Material online and all code is publicly available on GitHub (https://github.com/KaneLab/Tazollae-selection).
