# Supplementary material for: Selection Maintains Photosynthesis in a Symbiotic Cyanobacterium Despite Redundancy With its Fern Host
Source: Mol Biol Evol. 2025 Aug 6;42(8):msaf181. doi: 10.1093/molbev/msaf181 (PMC12362246; doi:10.1093/molbev/msaf181)
Supplement: msaf181_Supplementary_Data [file msaf181_supplementary_data.zip › Supplementary Materials_final.pdf]

Supplementary Materials

Supplementary Figures

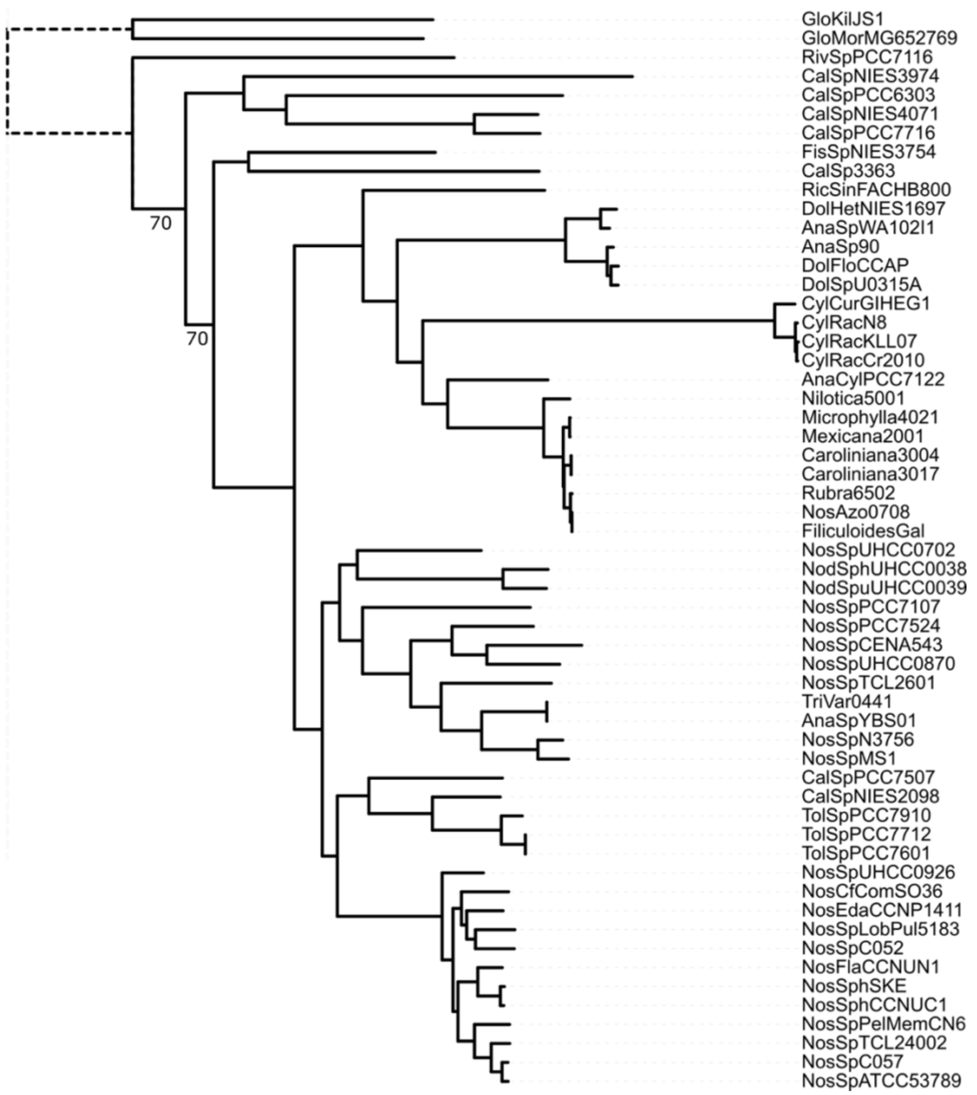

Supplementary Figure S1 – Phylogenomic placement of outgroup genomes and alternative phylogenomic tree construction using *RAxML-ng*

Phylogenomic tree generated by *RAxML-ng* using the same amino acid MSA of 1,131 orthogroups (with a minimum of 96.5% of species containing each orthogroup) that *OrthoFinder* gave to *FastTree* for the tree inference used in our analyses. At top, includes the seven deeply diverged Nostocales genomes that were removed from the main analyses as well as the two *Gloeobacter* genomes that were used to root the tree. Dotted lines indicate where branches between the Nostocales and *Gloeobacter* were shortened to improve visual resolution of shorter branches. Bootstrap support 100/100 for all nodes except where indicated. Ignoring branch lengths, this tree is identical to the *FastTree*-generated tree in terms. *RELAX* is the only analysis in which branch lengths might matter, but *RELAX* optimizes branch lengths internally, so this tree should have given identical results if substituted for the *FastTree* tree.

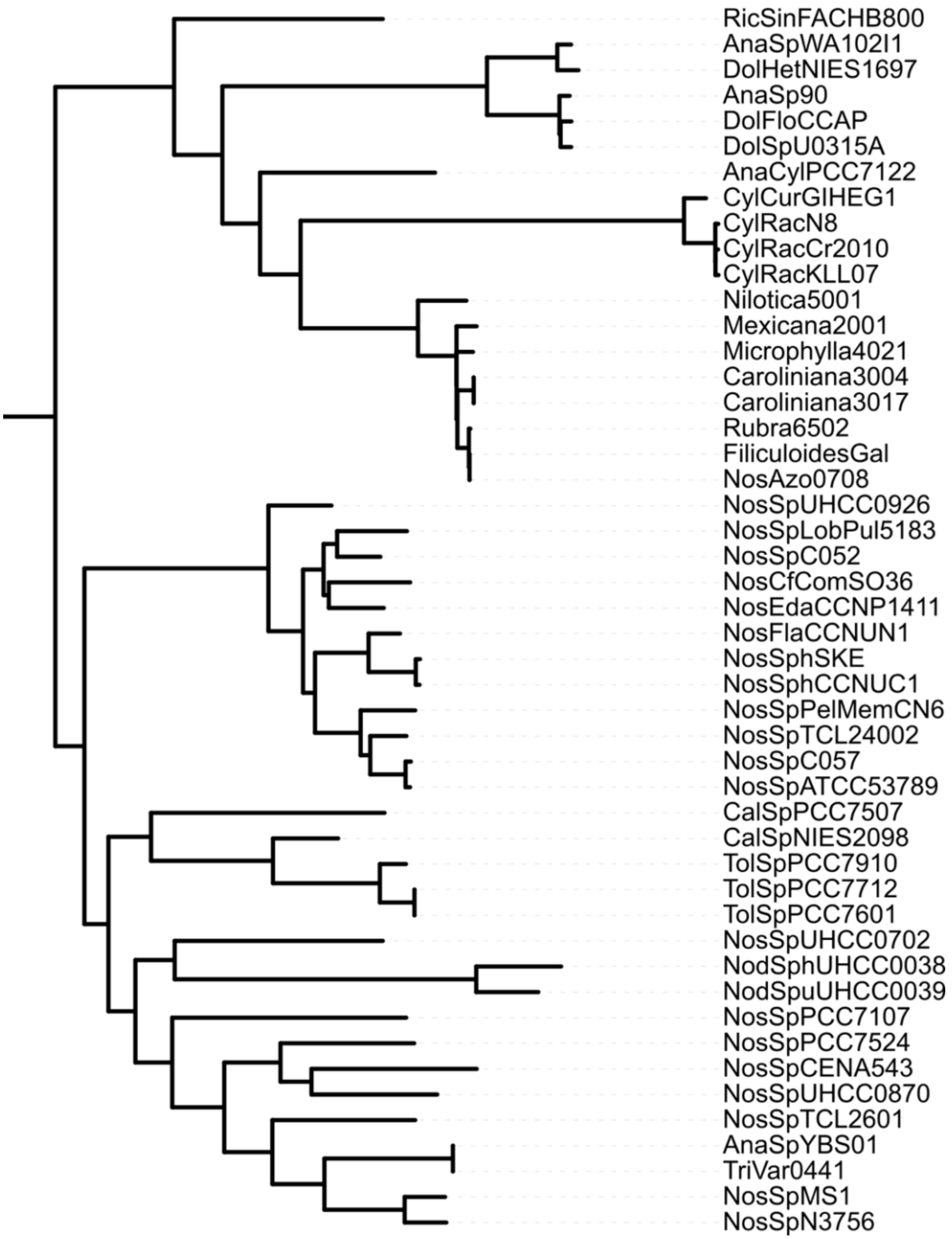

Supplementary Figure S2 – Alternative phylogenomic tree construction using *GTDB-tk*
Phylogenomic tree generated by *GTDB-tk* implemented in *KBase*. Bootstrap support lost in pruning of the larger *GTDB-tk* tree down to only the genomes used in the main analyses. The topology of this tree is different from those generated by *FastTree* in *OrthoFinder* and by *RAxML-ng*, but we chose to use the *FastTree* tree as described in "**Comparison of alternative methods for phylogenomic tree construction**" below.

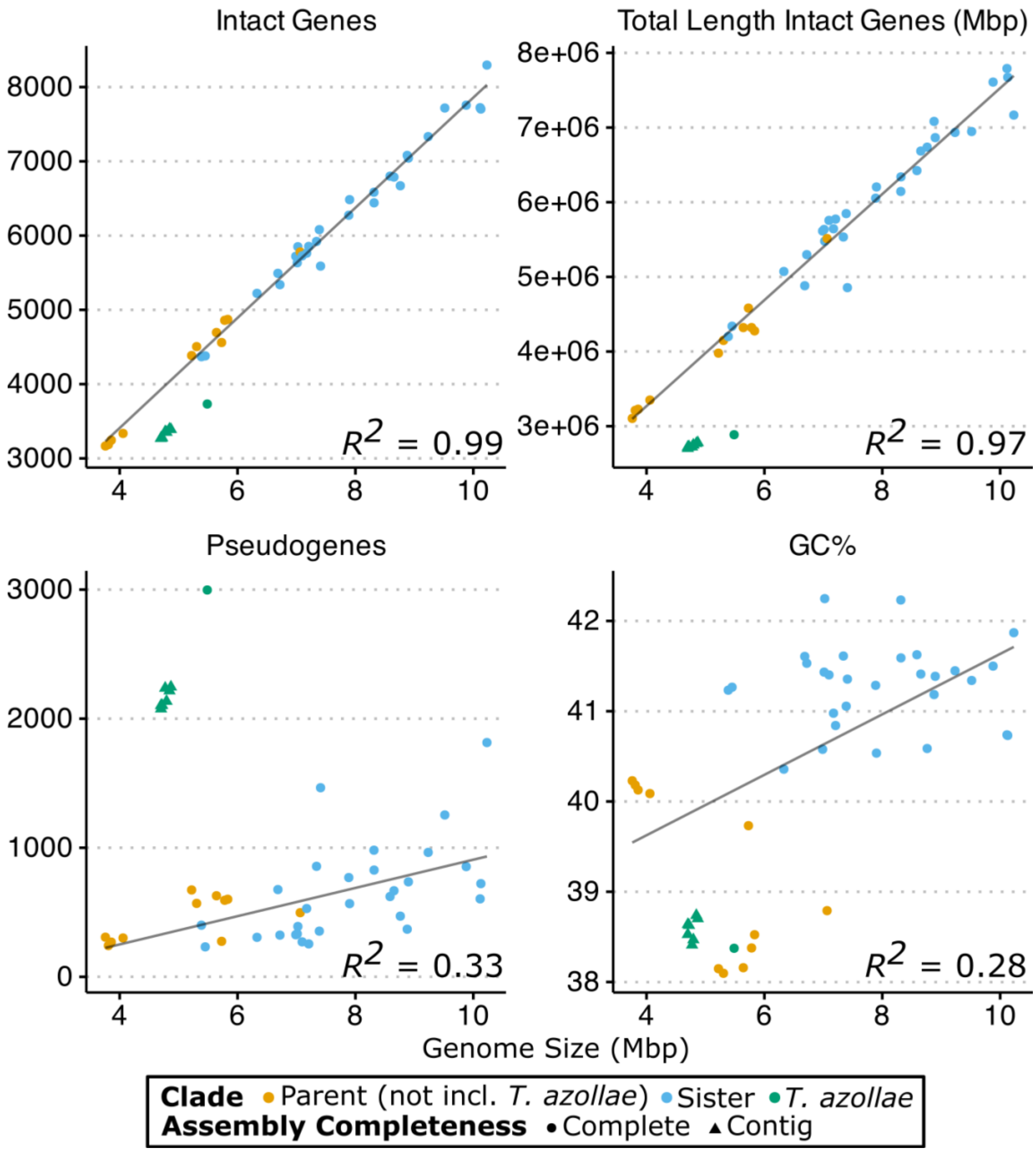

Supplementary Figure S3 - Genome statistics trends by genome size: Genome statistics for 40 complete Nostocales genomes, one complete *T. azollae* genome, and seven *T. azollae* MAGs. Trend lines and R^2 values were calculated without the *T. azollae* values using the *R* packages *ggplot2* and *ggpubr*. Clades and colors are as labeled in *Figure 1*.

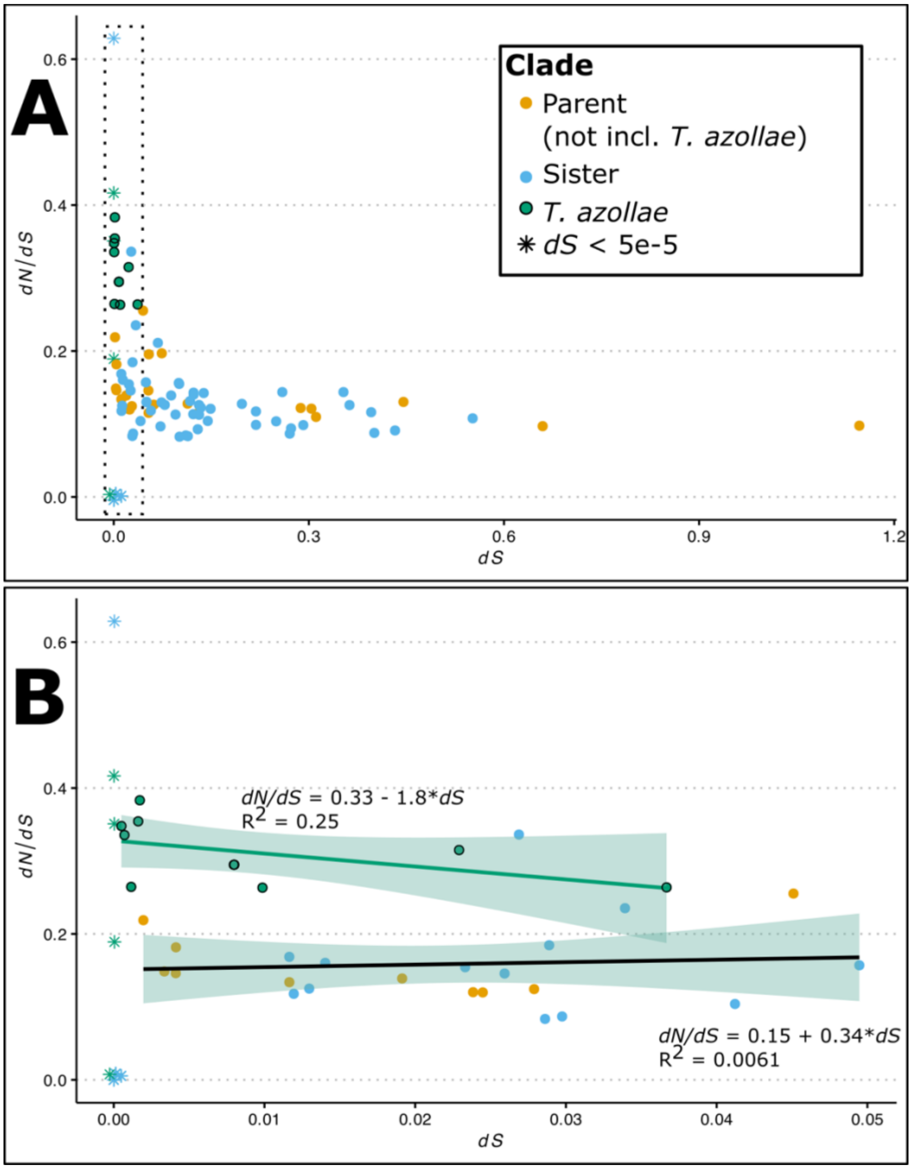

Supplementary Figure S4 – *dN/dS* by *dS*: Each point represents a branch on the phylogenomic tree in *Figure 1*. Asterisks are used to emphasize that we are not confident in interpreting *dN*/*dS* calculations when *dS* is very low (asterisks *dS*<5e-5, all other branches have 5.1e-3≤*dS*≤1.1). Panel B is an expanded view of the dotted inset in Panel A (*dS*<0.05). Trendlines with 95% confidence intervals for *T. azollae* and for free-living (Sister clade plus Parent clade, not including *T. azollae*) calculated using *geom_smooth()* function from *ggplot2* package (Wickham 2016) in *R* (R core team 2023) using only the points in Panel B (*dS*<0.05), not including those with *dS*<5e-5. This analysis addresses concerns that *dN/dS* only appears elevated in the *T. azollae* relative to the free-living Nostocales because the *T. azollae* clade is less diverged and *dS* is generally lower within it (Wolf et al. 2009). Panel A very clearly recreates the findings of Wolf et al. 2009 with the mean and variance of *dN/dS* decreasing with increasing *dS*. However, the separation between *T. azollae* and the free-living Nostocales is still clear and significant when only comparably short *dS* branches are considered (Panel B): the 95% confidence intervals are very nearly non-overlapping, and, not including branches with *dS*<5e-5, only one free-living branch has a *dN/dS* value greater than any *T. azollae* branch. The trendline slopes and low R^2 values indicate that there is not a significant negative trend in *dN*/*dS* over *dS* at these low *dS* values.

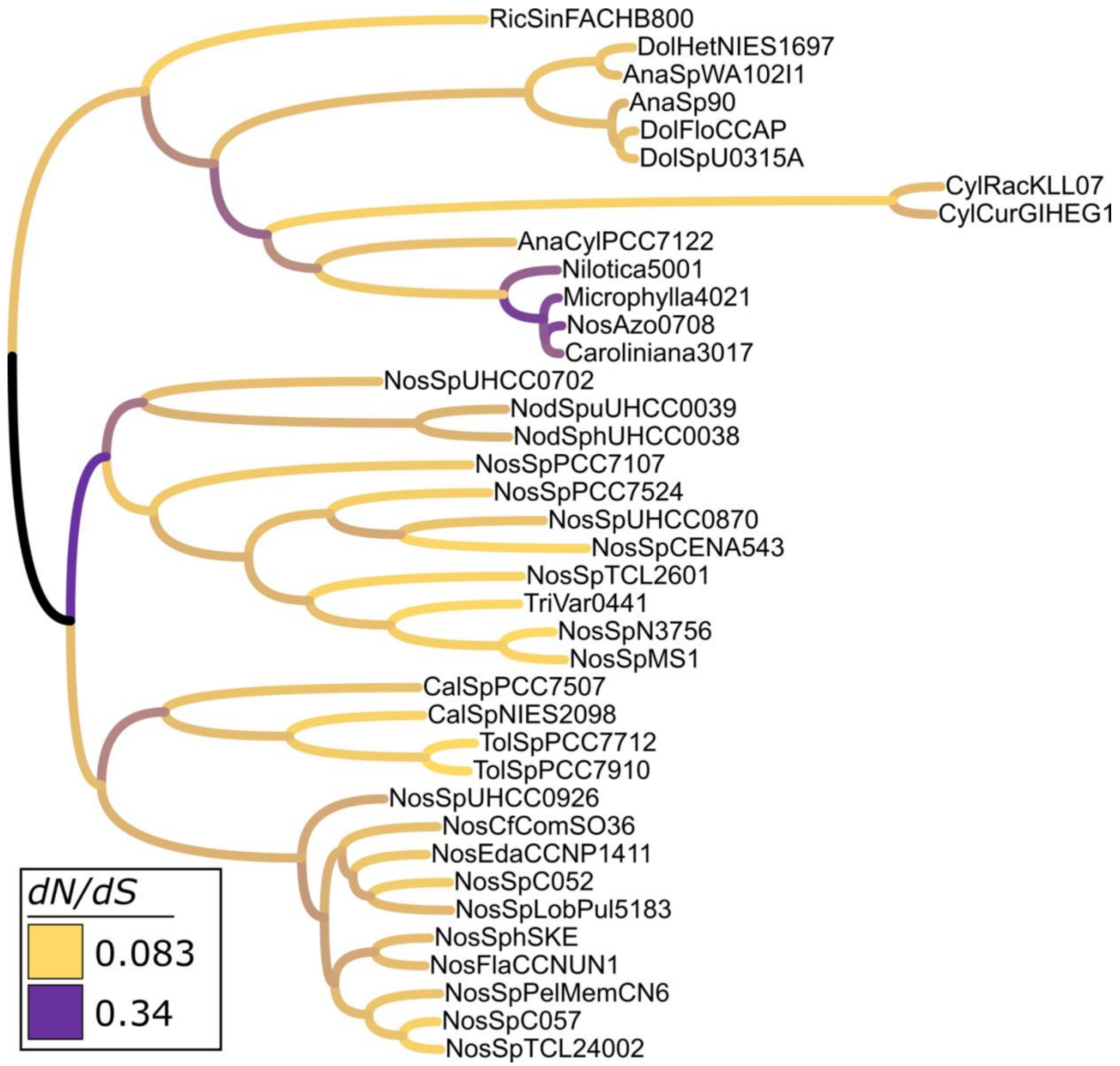

Supplementary Figure S5 – Genome-wide *dN/dS* on phylogenomic tree with low-divergence genomes removed: Phylogenomic tree as described in *Figure 1*, with genomes removed so that each pairwise distance between genomes *dS*>0.01. Branches are colored by *dN/dS* as calculated on this reduced genome set, using a concatenated alignment of 1,015 single-copy *OrthoFinder*-generated orthogroups.

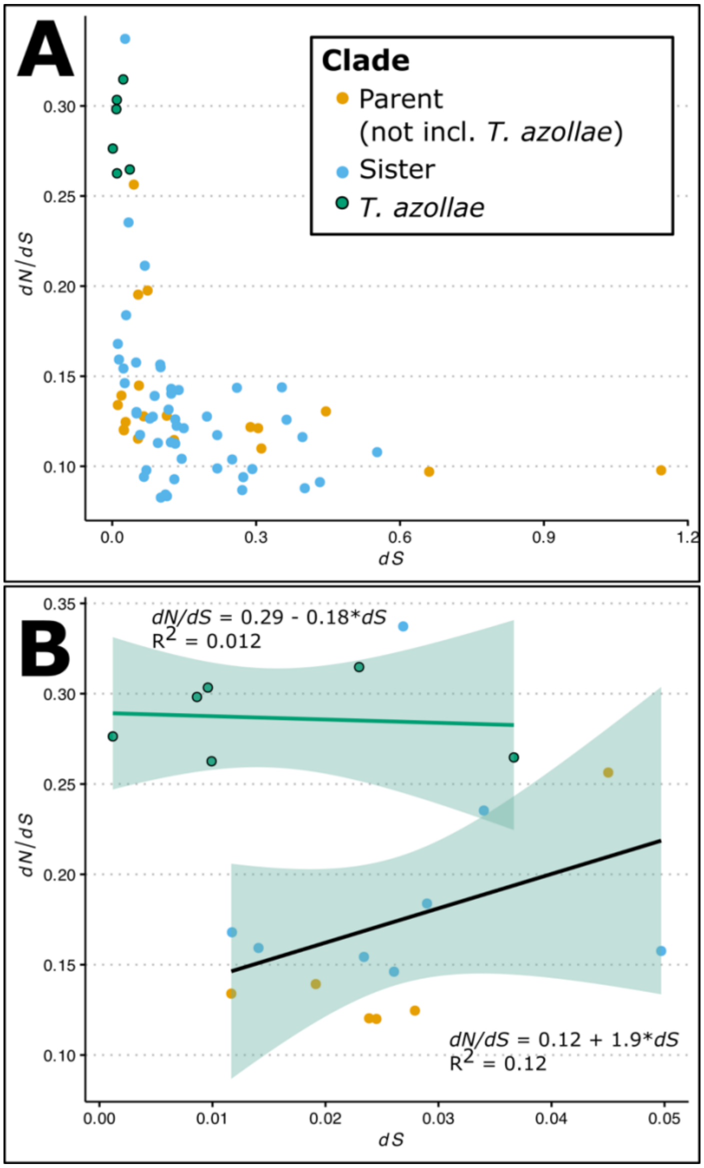

Supplementary Figure S6 – *dN/dS* by *dS* with low-divergence genomes removed: Each point represents a branch on the phylogenomic tree in *Figure S5*, which was generated by removing genomes so that each pairwise distance between genomes has *dS*>0.01. Panel B is an expanded view of the dotted inset in Panel A (*dS*<0.05). Trendlines with 95% confidence intervals for *T. azollae* and for free-living (Sister clade plus Parent clade, not including *T. azollae*) calculated using *geom_smooth()* function from *ggplot2* package (Wickham 2016) in *R* (R core team 2023) using only the points in Panel B (*dS*<0.05), not including those with *dS*<5e-5. This analysis addresses concerns that *dN/dS* only appears elevated in the *T. azollae* relative to the free-living Nostocales because the *T. azollae* clade is less diverged and *dS* is generally lower within it (Wolf et al. 2009). Panel A very clearly recreates the findings of Wolf et al. 2009 with the mean and variance of *dN/dS* decreasing with increasing *dS*. However, the separation between *T. azollae* and the free-living Nostocales is still clear and significant when only comparably short *dS* branches are considered (Panel B): the 95% confidence intervals are very nearly non-overlapping, and, not including branches with *dS*<5e-5, only one free-living branch has a *dN/dS* value greater than any *T. azollae* branch. The trendline slopes and low R^2 values indicate that there is not a significant negative trend in *dN/dS* over *dS* at these low *dS* values.

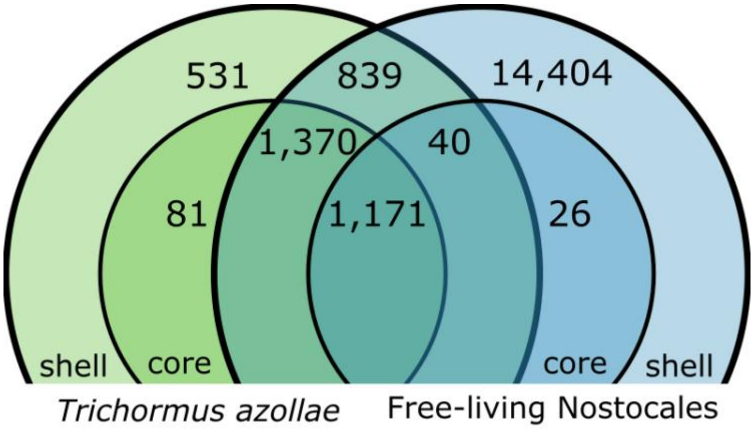

Supplementary Figure S7 – Venn diagram of orthogroups in the *T. azollae* and free-living Nostocales core and pan genomes as defined by orthogroup: Core orthogroups are intact in every genome. Shell orthogroups are intact in at least one genome. Pan genome is the union of the shell and core. Excludes predicted pseudogenes. Of 4,032 orthogroups in the *T. azollae* pan genome, 2,622 (65.0%) are in the *T. azollae* core. Of 1,410 orthogroups in the *T. azollae* shell genome, 418 (29.6%) are either only missing from *Nilotica5001* or only present in *Nilotica5001*. Of the 1,237 orthogroups that make up the free-living core genome, 66 (5.3%) are not in the *T. azollae* core genome (40 *shell*, 26 *absent*).

Supplementary Tables

In separate file, "Supplementary Tables.xlsx".

Supplementary Methods

prokka command:

with flags "*--compliant –rfam –kingdom Bacteria*"

***Pseudofinder* command:**

"*python pseudofinder.py annotate -t 20 -ce --use_deviation -l 0.65 --diamond -skpdb -hc 46 --genome $in_gbk --outprefix $query --database $db*"

OrthoFinder command:

"*orthofinder -y -M msa -o $outdir -f $indir*"

***HyPhy RELAX* orthogroup commands**

Orthogroups were initially submitted to *RELAX* using the command "*hyphy relax --alignment $msa_file --tree $tree_file --test "test" --reference "reference" --models Minimal --srv Yes --output $outfile > $stdoutfile*". If *RELAX* failed to converge, the orthogroup was re-run with the command "*hyphy CPU=1 relax --starting-points 100 --grid-size 2000 --models Minimal --srv Yes --alignment ${msa_dir}/${hog}.noStop.fa --tree ${tree_dir}/${hog}.labeled_tree.txt --test "test" --reference "reference" --output $outfile > $stdoutfile*". If *RELAX* still failed to converge, the orthogroup was removed from downstream analyses.

Mitigating effects of incomplete MAGs

As part of the assembly of the seven *T. azollae* MAGs (Dijkhuizen et al. 2021), reads were aligned using *BWA aligner* (Li and Durbin 2009) to the *Azolla filiculoides* reference nuclear and chloroplast genomes (Li et al. 2018) and all reads that aligned were filtered out. In addition, contigs were filtered out if they were assigned "Streptophyta" by *CAT* (von Meijenfeldt et al. 2019). We believe that this may cause certain genes to be removed, in full or in part, from the MAGs. Indeed, anecdotally, we do find that some genes that are common to both the *T. azollae* and host chloroplast genomes appear to be pseudogenes in each of the MAGs because they are cut-off by the end of a contig. It is of course impossible to evaluate if such an occurrence is truly a pseudogene or a result of the incomplete assembly. Furthermore, we cannot know whether some genes are entirely missing due to the incomplete assembly. While we cannot fully remove this limitation without obtaining complete genome assemblies, we hoped to improve our determinations that genes were present, absent, or pseudogenic by re-assembling the MAGs with the complete *Nostoc azollae* 0708 cyanobiont genome as a reference guide.

Raw metagenomic reads of the seven *Azolla* strains from which the seven *T. azollae* MAGs were derived were downloaded from the European Nucleotide Archive (accession PRJEB45214). The plant nuclear and chloroplast genomes were downloaded from fernbase.org. The *Nostoc azollae* 0708 (*NosAzo0708*) genome was already downloaded as described earlier. Reads were trimmed with *trimmomatic v. 0.39* (Bolger et al. 2014). Trimmed reads were aligned to the plant nuclear, plant chloroplast, and cyanobiont genomes using *bwa mem v. 0.7.17-r1188* (Li 2013). Any read pair for which at least one partner had its best alignment to the *NosAzo0708* genome, and neither partner aligned best to the plant nuclear or chloroplast genome, was kept. All other read pairs were removed. Assembly was done with *SPAdes v. 3.15.5*, with the "*--meta*" flag (Nurk et al. 2017). The same *prokka*, *Pseudofinder*, *OrthoFinder* pipeline described in the "*Initial Pipeline*" section of the methods was then re-run, replacing the seven MAGs with their re-assembled counterparts. This was all repeated in parallel using the "--*isolate*" flag of *SPAdes*. The results of this pipeline were then used to improve the present/absent/pseudogene matrices for the MAGs from the main pipeline. To this end, orthogroups from the different pipelines were matched if they contained exactly the same set of *NosAzo0708* loci. A predicted-intact gene in either of the matched orthogroups from the 2 re-assemblies meant that the corresponding main pipeline orthogroup would be updated to "intact" if it were not already. Likewise for pseudogenes unless the main pipeline orthogroup already contained an "intact" locus. Finally, a custom *BASH* script was used to determine which pseudogenes occurred at the ends of contigs and thus could not confidently be called pseudogenes. If whichever of the three corresponding orthogroups contained the most pseudogenes contained at least one end-of-contig pseudogene, then that main pipeline orthogroup was updated to "intact" to be as conservative as possible in pseudogene prediction.

This reassembly pipeline identified an additional 623 *T. azollae* loci as possibly intact (2.3% of the initial number of predicted intact loci) which were assigned to 270 (6.7%) of the orthogroups that contain at least one locus from at least one *T. azollae* genome. Other than the complete reference genome which had only 1 locus affected, the *T. azollae* genomes generally had fewer loci affected by this pipeline the further from the reference genome they are phylogenomically (*NosAzo0708*: 1, *Nilotica5001*: 58, *Mexicana2001*: 78, *Microphylla4021*: 81, *Caroliniana3004*: 96, *Rubra6502*: 97, *Caroliniana3017*: 98, *FiliculoidesGal*: 114). These possibly intact loci were not included in strength of selection analyses but were considered in analyses of gene presence/absence/pseudogenization. After this pipeline, only six orthogroups are intact in *NosAzo0708* but in zero of the *T. azollae* MAGs which increases our confidence that the MAG gene inventories are nearly complete. Without the re-assembly pipeline described in *Methods*, 25 orthogroups would be intact in *NosAzo0708* but zero *T. azollae* MAGs.

Genome-wide statistics increase our confidence that our incomplete assemblies should have minimal effects on our downstream analyses, particularly if we focus on the intact gene sets as opposed to pseudogene inventories. However, we cannot be confident that genes with close homologs in chloroplasts are missing from a contig-level assembly if they are present in the complete reference genome. *NosAzo0708* has a larger genome size, number of intact genes, total coding length, and number of

pseudogenes than the seven contig-level *T. azollae* MAGs. *NosAzo0708* also has the lowest GC% (*Figure 1B*). This is not surprising as it is the only complete *T. azollae* genome in our analysis. The fact that the total length of intact genes shows the least discrepancy, while the number of pseudogenes shows the greatest discrepancy, suggests to us that much of the sequence lost in the contig-level assemblies is non-coding, pseudogenes, or transposable elements. The *T. azollae* genome is full of transposons and other repetitive sequences (Ran et al. 2010) which can create pseudogenes and be difficult to assemble, so it makes sense that such regions would be more likely to be missing from the contig-level assemblies. The fact that *NosAzo0708* has the lowest GC% of the *T. azollae* further supports the idea that much of this missing sequence might be AT-rich "junk" DNA. For 52 orthogroups, *NosAzo0708* has more intact loci than any of the contig-level *T. azollae* MAGs. These 52 orthogroups account for 266-295 of the 337-462 "extra" intact ORFs in *NosAzo0708*. The annotations for those 52 orthogroups include 19 poorly annotated, 21 likely transposases, 4 photosynthesis-related genes, and 8 others. The presence of photosynthesis genes in this orthogroup set is likely due to the fact that reads were filtered by aligning to the *Azolla filiculoides* chloroplast genome before assembly (Dijkhuizen et al. 2021).

Methods Sensitivity

Comparison to *T. azollae* MAGs assembled from Li et al, 2018, *Nature Plants*

Initially, an additional six *T. azollae* MAGs were included in this analysis. These came from metagenomic data generated by Li et al. 2018. We assembled MAGs using methods as close as possible to those used by Dijkhuizen et al. 2021. All six host azolla strains sequenced by Li et al. 2018 were also sequenced in the Dijkhuizen et al. 2021 data set, though they were sequenced separately from separate individual plants. We found that corresponding *T. azollae* strains from the two sources overlapped in orthogroup content by 98.0-99.4% and that for orthogroups for which there was exactly one locus in each MAG from a given pair, the nucleotide sequences were identical for 99.5-99.8% of orthogroups. We removed the MAGs assembled from the data of Li et al.'s because we did not want such different scales of evolutionary divergence in the *T. azollae* compared to in the free-living Nostocales. These high rates of similarity also suggest that our findings are robust to population-level genomic differences.

Comparison of alternative methods for phylogenomic tree construction

Due to the importance of the phylogenomic tree to all of our analyses, we wanted to confirm the topology generated by *FastTree v. 2.1.11* (Price et al. 2010) implemented in *OrthoFinder v. 2.5.5* (Emms and Kelly 2019). To that end, we inferred trees using three other alternative methods, with resultant trees displayed in *Figure S1* and *Figure S2*:

(1) *RAxML-ng v. 1.2.0* (Kozlov et al., 2019) on the same amino acid MSA that *OrthoFinder* fed to *FastTree* for the tree inference used in our analyses. Command: "raxml-ng --all --msa $msa_from_orthofinder.faa --model LG+G8+F --tree pars{10} --bs-trees 100 --threads 36"

(2) *GTDB-tk v2.3.2* (Chaumeil et al. 2022), which uses *FastTree v. 2.1.10* implemented in *KBase v. 1.4.0* (Arkin et al. 2018; Chivian et al. 2023), on the full genomes used in our analyses as well as a large set of other genomes that *GTDB-tk* has curated from NCBI.

Ignoring branch lengths, the tree generated by *RAxML-ng* is identical to the tree generated by *FastTree* that was used in our analyses (*Figure 1*, *Figure S1*). The tree generated by *GTDB-tk* disagrees with the placements of *AnaCylPCC7122*, *NosSpUHCC0926*, *NosCfComSO36*, clade SB, and *Mexicana2001*. All trees have high bootstrap support (*Figure 1*, *Figure Ss*). Bootstrap support information was lost when pruning the *GTDB-tk* tree to show only genomes involved in this analysis. Because the *OrthoFinder*-generated MSA contains 1,131 orthogroups (with a minimum of 96.5% of species containing each orthogroup), while *GTDB-tk* uses only 120 genes, we believe that the *OrthoFinder*-generated MSA is likely to be more accurate. While *RAxML-ng* may be more reliable than *FastTree* in terms of branch lengths (Young et al. 2022), the only downstream analyses that uses branch

lengths are the *RELAX* analyses, but *RELAX* optimizes branch lengths using its own algorithm, so input branch lengths do not matter. We thus use the *FastTree* tree because using the *RAxML-ng* tree should not have changed any results, and we did not generate the *RAxML-ng* tree until after many other analyses had been performed.

Comparison of ORF annotation methods (*prokka* vs. RefSeq)

We used *prokka* to predict ORFs in all genomes including RefSeq genomes for consistency. We compared the outputs of these different annotation methods by running a parallel pipeline of pseudogene prediction by *Pseudofinder* and orthogroup prediction by *OrthoFinder* using none of the MAGs, but using all of the RefSeq genomes with ORFs as predicted on RefSeq as well as the same genomes with ORFs instead predicted by *prokka*. Across the 40 free-living Nostocales genomes and one *T. azollae* (*NosAzo0708*) genome from RefSeq, we find that if a locus from the RefSeq annotations is predicted intact and assigned to a given orthogroup, there is a mean 97.6% (min=95.7, med=97.7, max=98.8) chance that a locus from the *prokka* annotations is also predicted intact and assigned to that orthogroup. A mean of 84.8% of loci are in orthogroups with exactly one locus from each genome so loci can be directly compared. Of these, a mean of 90.9% (min=88.8, med=91.2, max=92) have identical start and stop positions and a mean of 98.1% (min=97.5, med=98.2, max=98.8) overlap ≥90% between the *prokka* and RefSeq ORF predictions. Thus, we are confident that choosing to use *prokka* to predict ORFs instead of using those provided by RefSeq should not significantly change our results but does provide consistency.

Comparison of pseudogene annotation methods (*Pseudofinder* vs RefSeq)

While ORF-prediction does not differ greatly between *prokka* and RefSeq, pseudogene prediction by *Pseudofinder* does differ from that of RefSeq. The *prokka*-*Pseudofinder* pipeline produced a median and mean of 2.8x as many pseudogenes as are annotated on RefSeq. It appears that nearly all of that difference is from the use of *Pseudofinder*, not the use of *prokka*, as *Pseudofinder* finds a mean and median of 2.6x as many pseudogenes in the RefSeq-derived ORFs as are annotated as pseudogenes on RefSeq. Furthermore, despite having more ORFs initially predicted by *prokka* than by RefSeq, the *prokka-Pseudofinder* pipeline predicts 17% fewer (3,732 compared to 4,495) intact protein-coding genes for *NosAzo0708* than does RefSeq. However, Ran et al 2010's count of intact genes (3,668) is within 2% of ours. Looking at the 40 free-living genomes, the absolute value of the difference in the number of intact protein coding genes has a mean of 2.0% (min=0.1, med=1.8, max=5.5), with 36 out of 40 having fewer intact genes predicted by our pipeline. It is possible that the discrepancy is so much larger in *NosAzo0708* due to some characteristic of the endosymbiont genome compared to free-living genomes that might make them more sensitive to method and parameters used for pseudogene prediction. As the boundaries of a pseudogene are not well defined, and their prediction depends on arbitrary parameters, we do not think these discrepancies suggest that our methods are not performing well. They are a reminder of the fallibility of pseudogene prediction and to be wary of counting pseudogenes.

Pseudogene assignment to orthogroups

Assigning pseudogenes to orthogroups can be difficult as we believe the unconstrained evolution of pseudogenes might break some of the assumptions built into programs such as *OrthoFinder*. We chose to use the top *blast* hit for assigning pseudogenes to orthogroups. We found that assignments were similar, but not identical, when pseudogenes were assigned based on the majority of *blast* hits. Of all pseudogenes, 99.2% had at least one *blast* hit; of those, 88.2% hit the same orthogroup for their top two hits, and 71.2% hit the same orthogroup for the majority of hits as for the top hit.

References

Arkin, Adam P., et al. "KBase: The United States Department of Energy Systems Biology Knowledgebase." *Nature Biotechnology*, vol. 36, no. 7, Aug. 2018, pp. 566–69. *DOI.org (Crossref)*, https://doi.org/10.1038/nbt.4163.

Bolger, Anthony M., et al. "Trimmomatic: A Flexible Trimmer for Illumina Sequence Data." *Bioinformatics*, vol. 30, no. 15, Aug. 2014, pp. 2114–20. *DOI.org (Crossref)*, https://doi.org/10.1093/bioinformatics/btu170.

Chaumeil, Pierre-Alain, et al. "GTDB-Tk v2: Memory Friendly Classification with the Genome Taxonomy Database." *Bioinformatics*, edited by Karsten Borgwardt, vol. 38, no. 23, Nov. 2022, pp. 5315–16. *DOI.org (Crossref)*, https://doi.org/10.1093/bioinformatics/btac672.

Chivian, Dylan, et al. "Metagenome-Assembled Genome Extraction and Analysis from Microbiomes Using KBase." *Nature Protocols*, vol. 18, no. 1, Jan. 2023, pp. 208–38. *DOI.org (Crossref)*, https://doi.org/10.1038/s41596-022-00747-x.

Dijkhuizen, Laura W., et al. "Far-Red Light-Induced Azolla Filiculoides Symbiosis Sexual Reproduction: Responsive Transcripts of Symbiont Nostoc Azollae Encode Transporters Whilst Those of the Fern Relate to the Angiosperm Floral Transition." *Frontiers in Plant Science*, vol. 12, Aug. 2021, p. 693039. *DOI.org (Crossref)*, https://doi.org/10.3389/fpls.2021.693039.

Emms, David M., and Steven Kelly. "OrthoFinder: Phylogenetic Orthology Inference for Comparative Genomics." *Genome Biology*, vol. 20, no. 1, Dec. 2019, p. 238. *DOI.org (Crossref)*, https://doi.org/10.1186/s13059-019-1832-y.

Kozlov, Alexey M., et al. "RAxML-NG: A Fast, Scalable and User-Friendly Tool for Maximum Likelihood Phylogenetic Inference." *Bioinformatics*, edited by Jonathan Wren, vol. 35, no. 21, Nov. 2019, pp. 4453–55. *DOI.org (Crossref)*, https://doi.org/10.1093/bioinformatics/btz305.

Li, Fay-Wei, et al. "Fern Genomes Elucidate Land Plant Evolution and Cyanobacterial Symbioses." *Nature Plants*, vol. 4, no. 7, July 2018, pp. 460–72. *DOI.org (Crossref)*, https://doi.org/10.1038/s41477-018-0188-8.

Li, Heng. *Aligning Sequence Reads, Clone Sequences and Assembly Contigs with BWA-MEM*. arXiv, 2013. *DOI.org (Datacite)*, https://doi.org/10.48550/ARXIV.1303.3997.

Li, Heng, and Richard Durbin. "Fast and Accurate Short Read Alignment with Burrows–Wheeler Transform." *Bioinformatics*, vol. 25, no. 14, July 2009, pp. 1754–60. *DOI.org (Crossref)*, https://doi.org/10.1093/bioinformatics/btp324.

Nurk, Sergey, et al. "metaSPAdes: A New Versatile Metagenomic Assembler." *Genome Research*, vol. 27, no. 5, May 2017, pp. 824–34. *DOI.org (Crossref)*, https://doi.org/10.1101/gr.213959.116.

Price, Morgan N., et al. "FastTree 2 – Approximately Maximum-Likelihood Trees for Large Alignments." *PLoS ONE*, edited by Art F. Y. Poon, vol. 5, no. 3, Mar. 2010, p. e9490. *DOI.org (Crossref)*, https://doi.org/10.1371/journal.pone.0009490.

R Core Team. *R: A Language and Environment for Statistical Computing*. R Foundation for Statistical Computing, 2023, https://www.R-project.org/.

Ran, Liang, et al. "Genome Erosion in a Nitrogen-Fixing Vertically Transmitted Endosymbiotic Multicellular Cyanobacterium." *PLoS ONE*, edited by Niyaz Ahmed, vol. 5, no. 7, July 2010, p. e11486. *DOI.org (Crossref)*, https://doi.org/10.1371/journal.pone.0011486.

Von Meijenfeldt, F. A. Bastiaan, et al. "Robust Taxonomic Classification of Uncharted Microbial Sequences and Bins with CAT and BAT." *Genome Biology*, vol. 20, no. 1, Dec. 2019, p. 217. *DOI.org (Crossref)*, https://doi.org/10.1186/s13059-019-1817-x.

Wickham, H. *ggplot2: Elegant Graphics for Data Analysis*. Springer-Verlag New York. ISBN 978-3-319-24277-4, 2016, https://ggplot2.tidyverse.org.

Wolf, Jochen B. W., et al. "Nonlinear Dynamics of Nonsynonymous (dN) and Synonymous (dS) Substitution Rates Affects Inference of Selection." *Genome Biology and Evolution*, vol. 1, Jan. 2009, pp. 308–19. *DOI.org (Crossref)*, https://doi.org/10.1093/gbe/evp030.

Young, Colin, et al. "An Evaluation of Phylogenetic Workflows in Viral Molecular Epidemiology." *Viruses*, vol. 14, no. 4, Apr. 2022, p. 774. *DOI.org (Crossref)*, https://doi.org/10.3390/v14040774.
